# Multiple Origins of Bioluminescence in Beetles and Evolution of Luciferase Function

**DOI:** 10.1093/molbev/msad287

**Published:** 2024-01-04

**Authors:** Jinwu He, Jun Li, Ru Zhang, Zhiwei Dong, Guichun Liu, Zhou Chang, Wenxuan Bi, Yongying Ruan, Yuxia Yang, Haoyu Liu, Lu Qiu, Ruoping Zhao, Wenting Wan, Zihe Li, Lei Chen, Yuanning Li, Xueyan Li

**Affiliations:** Key Laboratory of Genetic Evolution & Animal Models, Kunming Institute of Zoology, Chinese Academy of Sciences, Kunming, Yunnan 650223, China; School of Ecology and Environment, Northwestern Polytechnical University, Xi’an, Shaanxi 710072, China; Key Laboratory of Genetic Evolution & Animal Models, Kunming Institute of Zoology, Chinese Academy of Sciences, Kunming, Yunnan 650223, China; Kunming College of Life Science, University of the Chinese Academy of Sciences, Kunming, Yunnan 650204, China; School of Ecology and Environment, Northwestern Polytechnical University, Xi’an, Shaanxi 710072, China; Key Laboratory of Genetic Evolution & Animal Models, Kunming Institute of Zoology, Chinese Academy of Sciences, Kunming, Yunnan 650223, China; Key Laboratory of Genetic Evolution & Animal Models, Kunming Institute of Zoology, Chinese Academy of Sciences, Kunming, Yunnan 650223, China; Key Laboratory of Genetic Evolution & Animal Models, Kunming Institute of Zoology, Chinese Academy of Sciences, Kunming, Yunnan 650223, China; Room 401, No. 2, Lane 155, Lianhua South Road, Shanghai 201100, China; Plant Protection Research Center, Shenzhen Polytechnic University, Shenzhen 518055, China; Key Laboratory of Zoological Systematics and Application, School of Life Science, Institute of Life Science and Green Development, Hebei University, Baoding 071002, China; Key Laboratory of Zoological Systematics and Application, School of Life Science, Institute of Life Science and Green Development, Hebei University, Baoding 071002, China; Engineering Research Center for Forest and Grassland Disaster Prevention and Reduction, Mianyang Normal University, 621000 Mianyang, China; Key Laboratory of Genetic Evolution & Animal Models, Kunming Institute of Zoology, Chinese Academy of Sciences, Kunming, Yunnan 650223, China; Key Laboratory of Genetic Evolution & Animal Models, Kunming Institute of Zoology, Chinese Academy of Sciences, Kunming, Yunnan 650223, China; School of Ecology and Environment, Northwestern Polytechnical University, Xi’an, Shaanxi 710072, China; School of Ecology and Environment, Northwestern Polytechnical University, Xi’an, Shaanxi 710072, China; Institute of Marine Science and Technology, Shandong University, Qingdao 266237, China; Key Laboratory of Genetic Evolution & Animal Models, Kunming Institute of Zoology, Chinese Academy of Sciences, Kunming, Yunnan 650223, China

**Keywords:** bioluminescence, multiple parallel origins, luciferases, luciferin-binding sites, enzymatic properties

## Abstract

Bioluminescence in beetles has long fascinated biologists, with diverse applications in biotechnology. To date, however, our understanding of its evolutionary origin and functional variation mechanisms remains poor. To address these questions, we obtained high-quality reference genomes of luminous and nonluminous beetles in 6 Elateroidea families. We then reconstructed a robust phylogenetic relationship for all luminous families and related nonluminous families. Comparative genomic analyses and biochemical functional experiments suggested that gene evolution within Elateroidea played a crucial role in the origin of bioluminescence, with multiple parallel origins observed in the luminous beetle families. While most luciferase-like proteins exhibited a conserved nonluminous amino acid pattern (TLA^346 to 348^) in the luciferin-binding sites, luciferases in the different luminous beetle families showed divergent luminous patterns at these sites (TSA/CCA/CSA/LVA). Comparisons of the structural and enzymatic properties of ancestral, extant, and site-directed mutant luciferases further reinforced the important role of these sites in the trade-off between acyl-CoA synthetase and luciferase activities. Furthermore, the evolution of bioluminescent color demonstrated a tendency toward hypsochromic shifts and variations among the luminous families. Taken together, our results revealed multiple parallel origins of bioluminescence and functional divergence within the beetle bioluminescent system.

## Introduction

Bioluminescence represents one of the most extraordinary evolutionary adaptations, spanning across various species from bacteria and fungi to the animal kingdom (Harvey 1952; Haddock et al. 2010; Idrees et al. 2020). Most notably, terrestrial bioluminescence is exemplified in beetles, specifically within the taxonomic superfamily Elateroidea, including fireflies (Lampyridae), Asian star worms (Rhagophthalmidae), American railroad worms (Phengodidae), Asian click-like beetles (Sinopyrophoridae), and click beetles (Elateridae) (Johnson 1952; Lawrence 2010; Bi et al. 2019; Kusy et al. 2021; Kundrata et al. 2022). Lampyridae, containing ∼2,000 species, exhibits near-global distribution, except for the Antarctic region. Rhagophthalmidae, a relatively small family of around 60 species, is known from the eastern Palearctic and Oriental realms, while Phengodidae, containing ∼240 species, is distributed across the Americas. Sinopyrophoridae is a monotypic family, recently erected to accommodate a specific Asian species. Within Elateridae, a large and widely distributed family of around 11,000 species and some 200 luminous species have been discovered on the South American continent and Oceania. While Cantharidae and Lycidae within Elateroidea are phylogenetically close to the aforementioned luminous families, no bioluminescent species have been documented within these 2 families.

Bioluminescent beetles produce light within the peroxisomes of photocytes located in morphologically diverse luminous organs (Buck 1948) via a shared luciferase (Luc)-luciferin system (Viviani 2002). Remarkably, the identical luciferin structure observed across all luminous beetles has not been detected in nonluminous insects (Oba, Shintan, et al. 2008), implying that its evolutionary origin may be consistent with the origin of bioluminescence. Beetle Lucs play dual roles, not only facilitating bioluminescence reactions but also the biosynthesis of luciferin (Zhang et al. 2020). The conservation of the Luc-luciferin bioluminescent system among different luminous beetle families appears somewhat paradoxical to the morphological diversity of their luminous organs (Buck 1948; Bi et al. 2019), raising the question of whether beetle bioluminescence evolved from single or multiple origins. As Darwin noted, “The presence of luminous organs in a few insects, belonging to different families and orders, offers a parallel case of difficulty” (Darwin 1872). Recent comparative genomic studies involving 4 fireflies and 1 luminous click beetle have provided support for the parallel origin of bioluminescence between the Lampyridae and Elateridae beetles (Fallon et al. 2018; Zhang et al. 2020). However, the lack of high-quality reference genomes for key families such as Rhagophthalmidae, Sinopyrophoridae, Cantharidae, Lycidae, and Elateridae has limited our understanding of the origin and evolution of bioluminescence in Elateroidea. Additionally, the complex and often disputed phylogenetic relationships among the lineages of luminous and closely related nonluminous families have further complicated these questions (Kundrata et al. 2014; Zhang, Che, et al. 2018; Douglas et al. 2021; Cai et al. 2022). For example, the monotypic Sinopyrophoridae family has alternatively been proposed to form a “lampyroid clade” with 3 luminous families—Lampyridae, Phengodidae, and Rhagophthalmidae (Bi et al. 2019; Kusy et al. 2021)—or regarded as a new subfamily within Elateridae (Bi et al. 2019; Douglas et al. 2021). The nonelaterid “lampyroid clade” has been positioned within the otherwise monophyletic Elateridae (Douglas et al. 2021), while the nonluminous family Cantharidae has been proposed to form a clade with Lampyridae (Kundrata et al. 2014) or to have diverged before Lycidae (Zhang, Che, et al. 2018). These contentious phylogenetic relationships within beetles have led to multiple hypotheses regarding the origins of bioluminescence (Kundrata et al. 2014; Kusy et al. 2021). A clear understanding of true phylogeny is essential to effectively investigate the genetic and molecular mechanisms underlying diverse phenotypic traits in biology (Jarvis et al. 2014; Chen et al. 2019).

Gene duplication and subsequent functional divergence are key processes of genetic innovation (Ohno 1970; Hughes 1994). Postduplication, one copy may lose its function through nonfunctionalization or retain its function via either neofunctionalization or subfunctionalization (Innan and Kondrashov 2010; Long et al. 2013). Neofunctionalization, in particular, involves the acquisition of a new function due to the accumulation of mutations in duplicated genes (Innan and Kondrashov 2010). This process may involve negative trade-offs between the original and new functions carried out by different gene copies (Kondrashov 2005; Khersonsky et al. 2006). Beetle Luc is thought to have originated from the acyl-CoA synthetase (ACS) superfamily (Viviani 2002; Fallon et al. 2018; Adams and Miller 2020; Oba et al. 2020). Studies on ancient firefly Lucs have suggested that the evolution of Luc aligns with the evolutionary “trade-off” model, balancing its original ACS function with the newly acquired bioluminescence function (Oba et al. 2020). However, whether this evolutionary pattern applies to beetles outside the Lampyridae family remains uncertain. Beetles display a wide evolutionary diversity in bioluminescence, marked by variations in color (from red to green), position (dorsal and ventral surfaces, thorax, and abdomen), shape, and developmental stage (Bessho-Uehara et al. 2017; Liu et al. 2019; Liu et al. 2020; He et al. 2021; Al-Handawi et al. 2022). Despite substantial achievements in cloning and sequencing, site-directed mutagenesis, and detection of bioluminescent properties and protein structure of Luc (Conti et al. 1996; Kutuzova et al. 1997; Carrasco-Lopez et al. 2018; Liu et al. 2019; Liu et al. 2020; He et al. 2021; Al-Handawi et al. 2022), our understanding of the origin and evolution of Luc genes across the entire phylogenetic context of elaterid beetles remains poor.

To explore the origin and evolutionary novelty of beetle bioluminescence, we investigated the reference genomes of 6 luminous (Lampyridae: *Vesta saturnalis* [Ves]; Rhagophthalmidae: *Menghuoius giganteus* [Sta]; and Sinopyrophoridae: *Sinopyrophorus schimmeli* [Ess]) and nonluminous species (Elateridae: *Sinelater perroti* [Spe]; Cantharidae: *Lycocerus yunnanus* [Msp]; and Lycidae: *Platerodrilus igneus* [Lpj]; supplementary fig. S1, Supplementary Material online). Together with previously published beetle genomes (Kuhn et al. 2013; Cunningham et al. 2015; Fallon et al. 2018; Wang et al. 2019; Herndon et al. 2020; Zhang et al. 2020; Parisot et al. 2021), we conducted phylogenetic analyses across luminous and nonluminous beetles to investigate the origin and evolution of bioluminescence under a robust phylogenetic context. Based on multiple sequence alignments, comparative analyses of 3D structures, and functional analyses of ancestral, extant, and artificially site-directed mutated Lucs, our study provides robust evidence supporting multiple evolutionary origins of bioluminescence and the vital role of luciferin-binding sites (LBSs) of Lucs.

## Results

### High-Quality Assemblies and Genomic Characteristics of Elateroidea Beetles

**Fig. 1.**
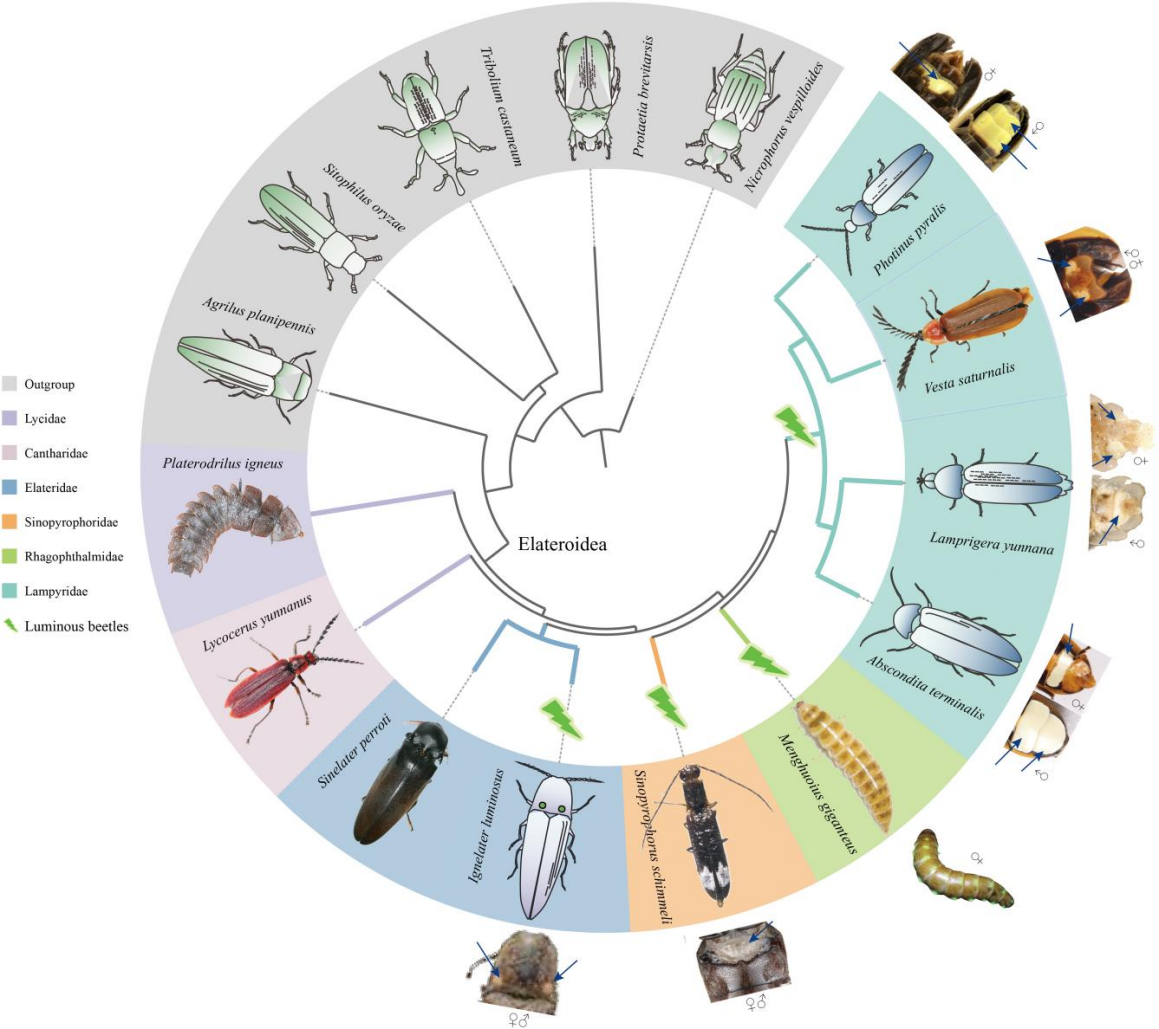
Phylogenetic relationships among luminous and nonluminous families in Elateroidea. Topology was recovered by concatenation based on data matrix #1 (AA: 348,711 amino acid sites) derived from 568 single-copy orthologous genes. All internal branches are supported with 100% ultrafast bootstrap values, except the ancestral branch of Elateridae (82%). Genomic data were collected from NCBI (schematics) and sequenced in this study (photos). For luminous species, body parts where light organs are located are shown with arrows, with pictures generated in this study or modified from Fallon et al (2018) and Zhang et al (2020).

Using Nanopore long-read and Illumina short-read sequencing technologies, we generated high-quality genome assemblies for 6 beetle species covering luminous and nonluminous lineages within Elateroidea (Table 1; Fig. 1; supplementary data S1 and S2, Supplementary Material online). All assembled genomes possessed high continuity and accuracy, as indicated by scaffold N50 length (2.5 to 21.0 Mb), Benchmarking Universal Single-Copy Orthologs (BUSCO) scores (94.7% to 97.8%), Illumina reads mapping ratios (95.7% to 98.8%), and transcript mapping ratios (87.4% to 94.2%) (Table 1). The assembled genome sizes ranged from 262.7 Mb (*M. giganteus*: Rhagophthalmidae) to 2,595.1 Mb (*P. igneus*: Lycidae) (Table 1), exhibiting ∼10-fold variation. Repeat elements constituted 38.51% to 72.24% of the investigated genomes (Table 1). In accordance with total genome size, the trilobite lycid beetle *P. igneus* contained the highest proportion of repeat elements (72.24%), while the rhagophthalmid beetle *M. giganteus* contained the lowest (38.51%). These variances in repeat elements contributed to the differences in overall genome size (Table 1). The annotated genes shared similar structures (i.e. mRNA length, coding sequence [CDS] length, intron length, exon length, and exon number) to those in published firefly genomes (supplementary fig. S2, Supplementary Material online; Fallon et al. 2018; Zhang et al. 2020). In addition, 82.07% to 90.07% of the predicted genes were supported by functional annotation in different databases (supplementary table S1, Supplementary Material online).

**Table 1:** Genome assembly and quality estimation of Elateroidea beetles

Family	Species	Genome assembly					Genome annotation		
		Assembly size (Mb)	Scaffold N50 (Mb)	Complete BUSCO ratio (%)	Illumina reads mapping ratio (%)	RNA-seq reads mapping ratio (%)	Repeat (% of genome)	Gene number	Complete BUSCO ratio (%)
Lycidae	*Platerodrilus igneus*	2,595.1	5.5	94.7	95.7	91.4	72.2	25,393	91.8
Cantharidae	*Lycocerus yunnanus*	375.1	2.7	94.9	97.3	87.4	51.4	18,811	90.4
Elateridae	*Sinelater perroti*	1,184.7	16.7	97.8	97.9	89.7	49.6	16,302	93.1
Sinopyrophoridae	*Sinopyrophorus schimmeli*	275.9	2.5	96.5	98.7	93.0	51.9	12,233	94.8
Rhagophthalmidae	*Menghuoius giganteus*	262.7	21.0	95.9	98.4	94.2	38.5	13,046	93.2
Lampyridae	*Vesta saturnalis*	2,054.2	2.8	96.3	98.8	87.5	58.1	22,921	93.2

### Phylogeny of Luminous and Nonluminous Beetle Lineages in Elateroidea

To clarify the relationships between luminous and nonluminous beetle lineages, we reconstructed phylogenetic trees using concatenation-based and coalescent-based approaches across 6 data matrices (Supplementary data S1, Supplementary Material online) encompassing 568 single-copy orthologous genes (supplementary fig. S3, Supplementary Material online: data matrices 1 to 3), 992/949 single-copy BUSCO genes (supplementary fig. S4a and b, Supplementary Material online: data matrices 4 to 5), and whole-genome alignments (WGAs; supplementary fig. S5, Supplementary Material online: data matrix 6). All trees showed consistent topology, except for the WGA trees. We applied DiscoVista to visualize and interpret gene tree discordance. Results indicated that the discordant topologies primarily involved species from the Cantharidae and Sinopyrophoridae families (supplementary fig. S6, Supplementary Material online), aligning with the inconsistent topologies derived from WGAs. Further Four-cluster likelihood mapping (FcLM) confirmed the sister position of Cantharidae to luminous families and the sister position of Sinopyrophoridae to Rhagophthalmidae + Lampyridae (supplementary figs. S7 to S10, Supplementary Material online). Taken together, we established robustly supported relationships among lineages of luminous beetles and their nonluminous relatives: (Lycidae + [Cantharidae + {Elateridae + (Sinopyrophoridae + [Rhagophthalmidae + Lampyridae])}]; Fig. 1).

### Changes in Genes in Elateroidea Beetles

**Fig. 2.**
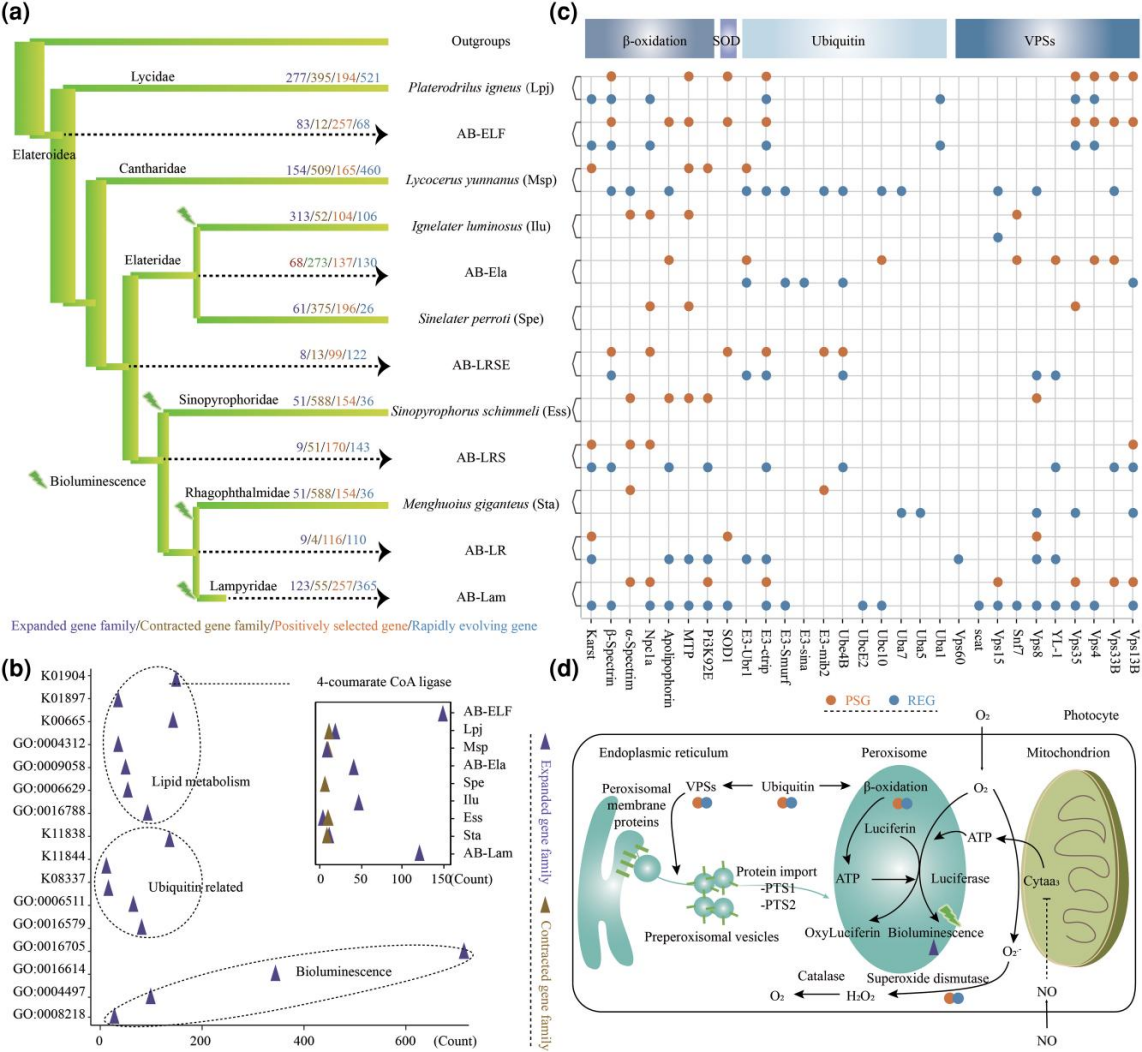
Significant changes in genes in Elateroidea beetles. a) PSGs, REGs, and expanded or contracted gene families are shown along the phylogenetic tree of Elateroidea. Dashed arrow represents common ancestral branch (AB-ELF) and internal ancestral branches (AB-Ela, AB-LRSE, AB-LRS, AB-LR, and AB-Lam) of Elateroidea. b) Enrichment of expanded or contracted gene families in common ancestral branch of Elateroidea (AB-ELF). Dotted line indicates expansion or contraction of 4-coumarate CoA ligase (K01904) throughout common ancestral and internal ancestral branches of Elateroidea (AB-ELF, AB-Ela, and AB-Lam) or species (Lpj, Msp, Spe, Ilu, Ess, and Sta) in Elateroidea. c) PSGs and REGs potentially related to bioluminescence in common ancestral and internal ancestral branches or species in Elateroidea corresponding to (a). YL-1, VPS-associated protein 72 homolog; Snf7, allelic to VPS32; Scat, scattered, VPS-associated protein 54; Ub, ubiquitin; Uba, ubiquitin-activating enzyme; Ubc, ubiquitin-conjugating enzyme; E3, E3 ubiquitin-protein ligase; SOD, superoxide dismutase; Pi3K92E, phosphatidylinositol 3-kinase 92E; MTP, microsomal triacylglycerol transfer protein; Npc1a,, Niemann-Pick type C-1a. d) Hypothetical pathway correlated with peroxisome biosynthesis and bioluminescence reaction in peroxisomes. Release of peroxisomal membrane proteins from endoplasmic reticulum to form preperoxisomal vesicles is mediated by ubiquitinated VPSs. These vesicles then undergo targeting and fusion with mature peroxisomes through PTS signaling (PTS1, C-terminal peroxisomal targeting signal 1, PTS2, N-terminal peroxisomal targeting signal 2). Following fusion, these vesicles divide to generate new peroxisomes (Dimitrov et al. 2013; Mast et al. 2018). Within newly formed peroxisomes, bioluminescence is released through a shared Luc-luciferin system (Viviani et al. 2022) in the presence of O_2_, Mg^2+^, and ATP. ATP is primarily generated through β-oxidation of fatty acids within peroxisomes (Wanders et al. 2001; Islinger et al. 2018) or transferred from mitochondria. Bioluminescence can interact with superoxide dismutase and catalase to mitigate oxidative imbalance (Bechara and Stevani 2018).

To investigate the genomic basis of bioluminescence origin in Elateroidea, we performed comparative genomic analyses of luminous and nonluminous beetles. We first analyzed gene families that expanded or contracted during the evolutionary process along the luminous and nonluminous lineages (Fig. 2a). Our data revealed that 83 expanded families (*P* < 0.05) within the common ancestral branch of Elateroidea (AB-ELF) were enriched in bioluminescence-related enzymes or processes, such as redox-related processes (bioluminescence, oxidoreductase activity, and monooxygenase activity) and lipid metabolism-related processes (fatty acid synthase, long-chain ACS, and 4-coumarate-CoA ligase [4CL]; Fig. 2b; supplementary data S3, Supplementary Material online). The 4CL family, which belongs to the ACS superfamily, together with the Luc and luciferase-like (LL) gene family (LLL; Zhang et al. 2020), was expanded in certain ancestral branches and internal branches/species of Elateroidea (AB-ELF, ancestral branch of Elateridae [AB-Ela], *Ignelater luminosus* [Ilu], and ancestral branch of Lampyridae [AB-Lam]) but both expanded and contracted in internal species (Lpj, Msp, Ess, and Sta; Fig. 2b; supplementary data S3, Supplementary Material online).

We also observed higher evolutionary rates in Elateroidea species than in the outgroup species (supplementary fig. S11, Supplementary Material online). We further identified rapidly evolving genes (REGs; supplementary data S4, Supplementary Material online) and positively selected genes (PSGs; supplementary data S5, Supplementary Material online) in the phylogenetic tree (Fig. 2a). Results indicated that the REGs and PSGs in most luminous species were enriched in processes associated with peroxisomal formation, metabolism, and oxidative stress tolerance (Fig. 2c; supplementary data S6 and S7, Supplementary Material online). Peroxisomes are key metabolic organelles in lipid metabolism, generating ATP through the β-oxidation of fatty acids (Wanders et al. 2001; Islinger et al. 2018). Moreover, the photocytes within peroxisomes are the exclusive sites where beetles emit light by transforming the chemical energy of ATP (Adams and Miller 2020). Vacuolar protein sorting (VPS) and associated proteins, as well as ubiquitin-related proteins, underwent significant changes in ancestral branches and internal branches/species of Elateroidea (Fig. 2c). Furthermore, our results showed that genes associated with lipid storage (e.g. *spectrin*, *karst*, *apolipophorin*) exhibited significant alterations (Fig. 2c). Superoxide dismutase, which cooperates with catalase and beetle bioluminescence to mitigate oxidative imbalance (Bechara and Stevani 2018), was also under positive selection in AB-ELF, the ancestral branch of Lampyridae + Rhagophthalmidae + Sinopyrophoridae + Elateridae (AB-LRSE), and the ancestral branch of Lampyridae + Rhagophthalmidae (AB-LR; Fig. 2c). Collectively, these findings suggest that the peroxisomal-related genes underwent substantial alterations within Elateroidea, potentially providing components for beetle bioluminescence, such as substrate transportation, energy provision, and intracellular redox homeostasis (Fig. 2d).

### Multiple Parallel Origins of Luc Genes in Different Luminous Beetle Families

To resolve the evolutionary origins of bioluminescence in beetles, we explored the evolutionary process of Luc within the ACS superfamily (supplementary data S8 and S9, Supplementary Material online), given its important role in D-luciferin biosynthesis and light emission (Zhang et al. 2020), and the expansion and contraction of 4CL within Elateroidea (Fig. 2a and b; supplementary data S3, Supplementary Material online). Phylogenetic analyses revealed a close relationship between the LLL and 4CL families (supplementary figs. S12 and S13 and data S8 and S9, Supplementary Material online). We then applied MIPhy to quantify phylogenetic instability (Curran et al. 2018). Results showed that 2 4CL and 2 LLL subclades (including Lucs from all luminous families) exhibited the highest instability scores (supplementary fig. S14A, Supplementary Material online). In addition, we employed PAML to calculate *Ka*/*Ks* ( $\underline{\omega }$) based on the number of synonymous and nonsynonymous substitutions for branches of the LLL subclade that contained Lucs under the free ratio model (model = 1, NS sites = 0). Results showed that the $\underline{\omega }$ values for all branches leading to Lucs were <1 (supplementary fig. S14B, Supplementary Material online), suggesting functional constraint on these Lucs. However, $\underline{\omega }$ values for branches leading to both *IluLuc* (Elateridae: 0.3342) and *EssLuc* (Sinopyrophoridae: 0.0545) were much higher than those from branches leading to the adjacent paralogs, i.e. *IluPACS10*/*IluPACS11* and *EssLuc-LL1*/*EssLuc-LL2*, respectively, suggesting that relaxation of functional constraint may have occurred for these 2 Luc genes. Although the $\underline{\omega }$ values for branches leading to *PpyLuc1* and *PpyLuc2* (Lampyridae: 0.0528 and 0.0850), and *StarLuc* (Rhagophthalmidae: 0.0722) were much <1, the value of their common ancestral branch was very large (>>1), indicating strong positive selection on their common ancestral gene. These findings suggest that the Luc genes in the LLL subclade may have undergone rapid evolution, resulting in 3 parallel origins of bioluminescence in Lampyridae–Rhagophthalmidae, Sinopyrophoridae, and Elateridae, respectively.

**Fig. 3.**
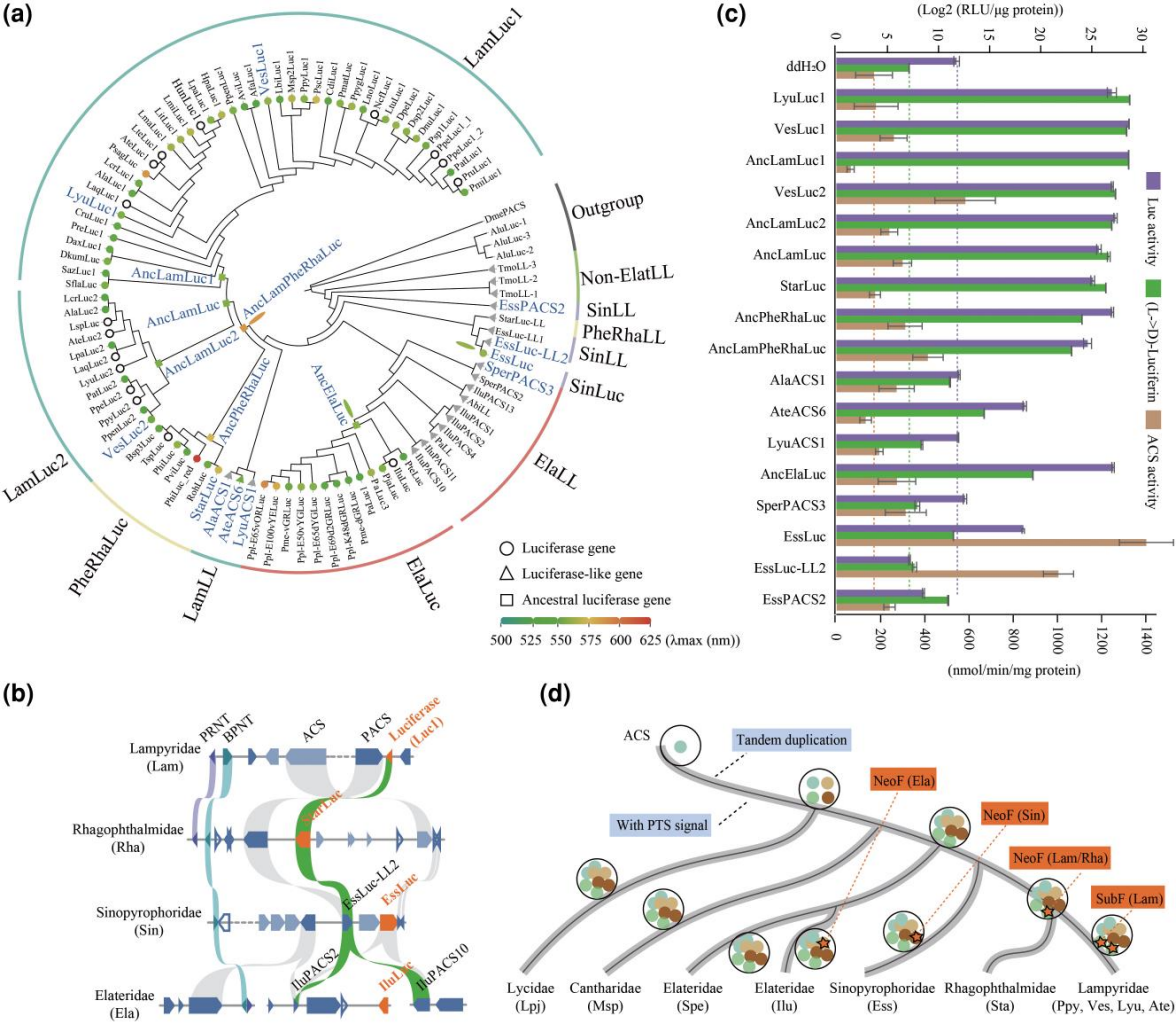
Multiple parallel origins of Luc in beetles. a) Phylogenetic tree of Luc and LL genes. Oval shapes indicate position of Luc genes. Gene names marked with larger size performed biochemical functional experiments in this study. Circles, triangles, and squares represent extant beetle Luc genes, LL genes, and ancestral Luc genes, respectively, and their colors represent corresponding maximum emissions (*λ*max). White circles and gray triangles represent the absence of gene spectrum detection or near-zero luminescence activity, respectively. Ancestral Luc gene reconstructed using PAML v4.9 with Jones matrix. b) Microsynteny analysis of syntenic block surrounding Lucs across 4 luminous families. Syntenic Luc is highlighted in green. c) Biochemical properties of reconstructed ancestral and extant LLL genes. Error bars represent standard error of the means from 3 independent replicates. d) Diagram of multiple parallel origins of beetle Lucs. Orange star indicates neofunctionalization of different copies of ACS to form Luc. NeoF, neofunctionalization; SubF, subfunctionalization; Elat: Elateroidea; Lpj, *Platerodrilus igneus* (Lycidae); Msp, *Lycocerus yunnanus* (Cantharidae); Spe, *Sinelater perroti* (Elateridae, Ela); Ilu, *Ignelater luminosus* (Elateridae, Ela); Ess, *Sinopyrophorus schimmeli* (Sinopyrophoridae, Sin); Sta, *Menghuoius giganteus* (Rhagophthalmidae, Rha); Ppy, *Photinus pyralis* (Lampyridae, Lam); Ves, *Vesta saturnalis* (Lampyridae, Lam); Lyu, *Lamprigera yunnana* (Lampyridae, Lam); Ate, *Abscondita terminalis* (Lampyridae, Lam); Phe, Phengodidae; PheRha: Phengodidae-Rhagophthalmidae; PRNT, Polyribonucleotide nucleotidyltransferase; BPNT, 3′(2′),5′-bisphosphate nucleotidase; PACS, ACS with peroxisomal targeting signal (PTS).

To determine the reliability of the above results, we employed GeneRax v2.0.4 to reconcile the gene family tree of the LLL subclade with the heterogeneous species trees. Regardless of the different species tree topologies (supplementary fig. S14c and d, Supplementary Material online), the Lucs consistently formed 3 clades in the reconciled gene family trees, notably, Lampyridae–Rhagophthalmidae, Sinopyrophoridae, and Elateridae (supplementary fig. S14e, Supplementary Material online). Furthermore, combining genome-wide identified Luc and LL genes (supplementary data S8 and S9, Supplementary Material online) with previously reported cloned Luc genes in luminous beetles (supplementary data S10, Supplementary Material online), we conducted phylogenetic analyses. Results showed that the Lucs from Lampyridae and Rhagophthalmidae/Phengodidae formed 1 terminal clade, while the Lucs from Elateridae and Sinopyrophoridae formed 2 distinct clades (Fig. 3a; supplementary fig. S15, Supplementary Material online). Interestingly, no Luc genes were found in the nonluminous beetles, i.e. *P*. *igneus* (Lycidae), *L. yunnanus* (Cantharidae), and *S*. *perroti* (Elateridae; Fig. 3a; supplementary fig. S15, Supplementary Material online). Furthermore, molecular clock analysis indicated that the origin times of the Luc genes in the different luminous families were variable (Lampyridae + Rhagophthalmidae/Phengodidae: 245.5 Ma; Sinopyrophoridae: 156.32 Ma; Elateridae: 181.38 Ma; supplementary fig. S15, Supplementary Material online).

Based on synteny analyses (Fig. 3b; supplementary fig. S16 and data S11 and S12, Supplementary Material online), 2 copies of firefly Luc genes (Lampyridae: *Luc1* and *Luc2*) exhibited a clear collinearity relationship, with *Luc1* displaying orthology to *StarLuc* in Rhagophthalmidae. However, *Luc1* did not show orthology to the extant Luc locus of Sinopyrophoridae (*EssLuc*) or Elateridae (*IluLuc*) but did display orthology to the tandem repeat LL locus of Sinopyrophoridae (*EssLuc-LL2*) and Elateridae (*IluPACS2* and *IluPACS10*). In Lampyridae species, *Luc2* is located on a different scaffold from *Luc1*, with no LL genes found in its vicinity, suggesting that *Luc2* underwent gene duplication and subsequent translocation. The divergence in the transcript expression patterns of *Luc2* shown in previous study (Bessho-Uehara et al. 2017) may have given rise to its retention by subfunctionalization. Luminescence activities were evaluated in the presence of D-luciferin, ATP, and Mg^2+^, revealing varying intensities among the Lucs (*VesLuc1*, *VesLuc2*, *StarLuc*, and *EssLuc*), except for LL genes (*AlaACS1*, *LyuACS1*, *SperPACS3*, *EssLuc-LL2*, and *EssPACS2*), which were at trace levels (Fig. 3c). Thus, we distinguished Luc genes from LL genes based on sequence similarity, gene tree analysis, and luminescence activity. For example, *SperPACS3*, sister to *SperPACS2* (Fig. 3a) in nonluminous species (*Sinelater perroti*: Elateridae), displayed no significant luminescence intensity (Fig. 3c) and was thus classified as a LL gene. *EssLuc*, present in luminous species (*S. schimmeli*: Sinopyrophoridae), displayed luminescence intensity/D-luciferin transformation ability (Fig. 3c) and was thus classified as a Luc gene, while other copies (*EssLuc-LL1*, *EssLuc-LL2*, and *EssPACS2*) were designated as LL genes because no luminescence intensity was detected (Fig. 3c).

Taken together, our comprehensive analysis integrating phylogenetic relationships, divergence times, syntenic analysis, and biochemical functional experiments provides compelling evidence to support the independent origins of Luc genes in Lampyridae–Rhagophthalmidae, Sinopyrophoridae, and Elateridae (Fig. 3d). In the nonluminous ancestor of Elateroidea, a tandem duplication event within the ACS gene on a single scaffold/chromosome led to the emergence of multiple ancestral ACS copies. Subsequently, these ACS copies underwent further evolutionary changes (expansion or contraction) in different families. Prior to the divergence of the Lampyridae and Rhagophthalmidae families, one of the ACS copies underwent neofunctionalization to become the ancestral Luc gene (Lam/Rha), which was duplicated again in Lampyridae, with one copy translocated in a separate scaffold/chromosome and subfunctionalized. Similarly, in Sinopyrophoridae and Elateridae, different copies of the ACS genes independently acquired Luc function through changes in their functional properties.

### Functional Evolution of Beetle Luc Proteins

To trace the functional evolution of beetle Lucs, we investigated the structural and functional characteristics of extant, ancestral, and artificially mutated Luc proteins. Using AlphaFold Colab (Mirdita et al. 2022), we first predicted the structures of the extant and reconstructed ancestral Luc proteins. Results showed that the N-terminal domains of all investigated Lucs were conserved, but their disordered loops and basic components of 5 β strands and 3 α helices at the C-terminal domain were folded into 2 distant conformations (supplementary fig. S17, Supplementary Material online). These predicted structures are consistent with the crystal structure of Luc in fireflies (*Photinus pyralis*; Conti et al. 1996). We verified the properties of the ancestral and extant Lucs and their close LL proteins, including luminescence intensity, L-luciferin to D-luciferin transformation ability, ACS activity, and bioluminescent spectra (Fig. 3a and c; supplementary figs. S18 and S19 and data S10, Supplementary Material online). We further conducted Pearson correlation analysis of luminescence intensities, L-luciferin to D-luciferin transformation abilities, and ACS activities of extant Lucs and reconstructed ancestral Lucs (supplementary fig. S18, Supplementary Material online). Results showed that ACS activities were negatively correlated with both luminescence intensities and L-luciferin to D-luciferin transformation abilities, while a positive correlation was observed between luminescence intensities and L-luciferin to D-luciferin transformation abilities. Regarding bioluminescent color (Fig. 3a; supplementary fig. S19 and data S10, Supplementary Material online), spectral experiments showed that AncLamPheRhaLuc emitted orange light (*λ*max = 589 nm), while AncPheRhaLuc (*λ*max = 575 nm), AncLamLuc (*λ*max = 559 nm), AncLamLuc1 (*λ*max = 562 nm), and AncLamLuc2 (*λ*max = 559 nm) exhibited hypsochromic shifts compared with AncLamPheRhaLuc. Furthermore, we observed a larger *λ*max (5 to 12 nm) and a shift toward shorter wavelengths in the luminescence spectrum, consistent with the findings reported by Oba et al (2020).

**Fig. 4.**
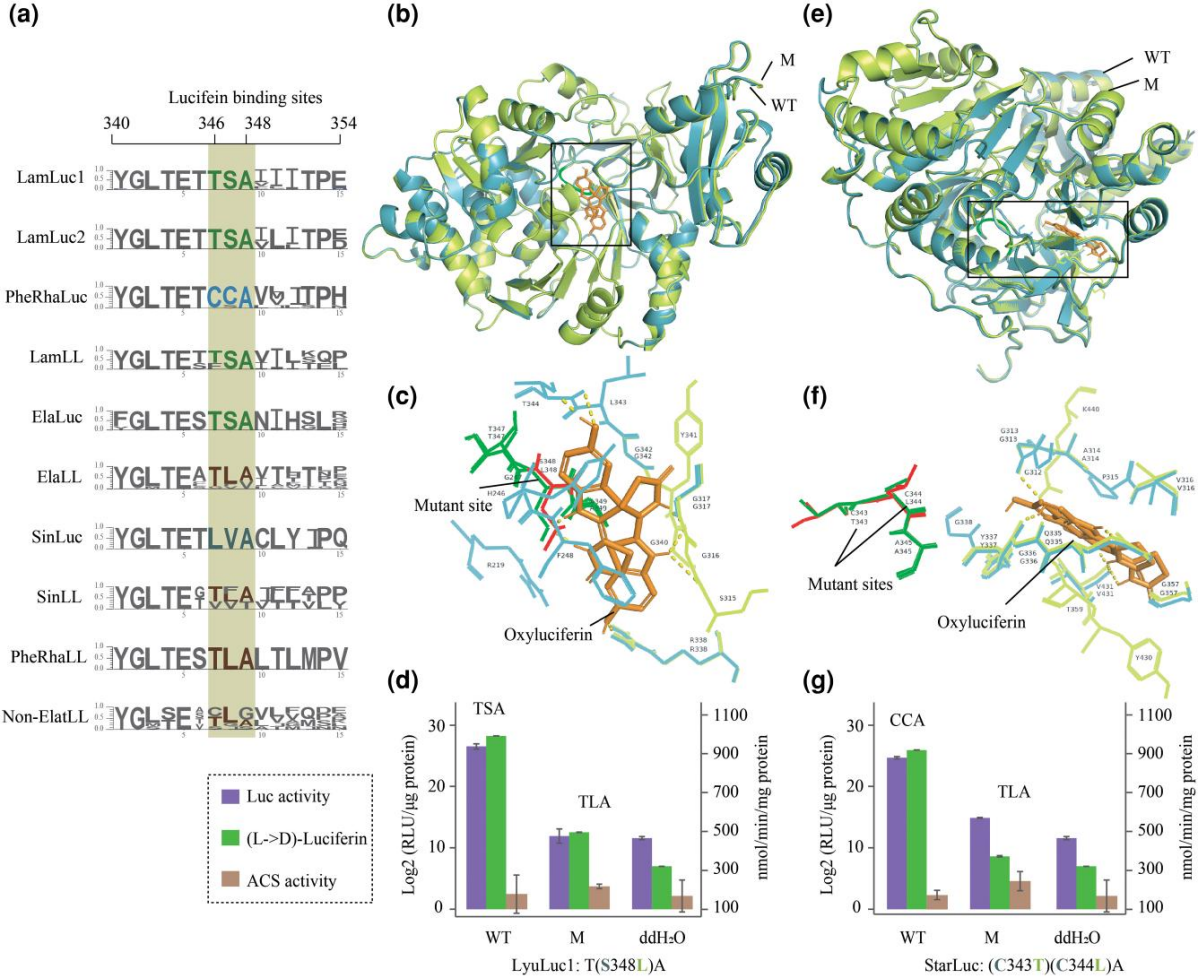
Functional evolution of LBS patterns of Luc and LL genes. a) Patterns of LBSs in LLL genes. Position of gene corresponds to Fig. 3a. b to d) Predicted 3D structure and biochemical properties of wild-type (WT) and artificially site-directed mutated (M) Luc-oxyluciferin complexes of *LyuLuc1*. e to g) Predicted 3D structure and biochemical properties of WT and M Luc-oxyluciferin complexes of *StarLuc*.

To investigate key Luc sites during functional evolution, we conducted multiple sequence alignments for Luc and LL genes. Results revealed distinct amino acid patterns at the LBSs (“346 to 348”) of Lucs in different families (luminous patterns: Lampyridae and Elateridae: TSA; Rhagophthalmidae/Phengodidae: CCA/CSA; Sinopyrophoridae: LVA) and nonluminous LL genes (nonluminous pattern: TLA; Fig. 4a; supplementary data S10, Supplementary Material online). The LBSs in the reconstructed ancestral Lucs were consistent with those of the corresponding extant Lucs (supplementary data S10, Supplementary Material online). In our previous study, we found similar LBS patterns (Zhang et al. 2020), but with no in-depth functional verification. In this study, we conducted site-directed mutagenesis for 2 extant Lucs, representing 2 luminous patterns (*LyuLuc1*: T(S348L)A and *StarLuc*: (C343T)(C344L)A; supplementary data S10, Supplementary Material online). Remarkably, the 3D conformations of the mutant and wild-type Lucs exhibited striking similarity, with a root mean square deviation (RMSD) ranging from 0.105 to 0.325 Å for 3,315 to 3,937 aligned atoms in the superposed structures (supplementary fig. S20, Supplementary Material online), suggesting that mutation of these residues in the disordered loop region does not change overall conformation. The conformations of the Luc-oxyluciferin compound between mutants and wild-types were nearly identical (Fig. 4b and e), suggesting that mutation of these residues does not impact overall conformation in reaction with their substrate. However, compared with the wild-types, the mutants exhibited considerable changes in their local environment, e.g. the range of oxyluciferin 3.5 Å (Fig. 4c and f; supplementary table S2, Supplementary Material online). The replacement of hydrophilic amino acid (C/S) with hydrophobic amino acid (L) resulted in looser molecular pockets (Fig. 4c and f). Furthermore, experimental data demonstrated a sharp decrease and near disappearance of luminescence intensity and L-luciferin to D-luciferin transformation activity (Fig. 4d and g) when the luminous patterns were replaced by nonluminous patterns. In addition, the original ACS activities of the 2 extant Luc mutants were slightly higher than the corresponding wild-types (Fig. 4d and g).

## Discussion

This study clarified the origin of Luc and bioluminescence in beetles based on high-quality reference genomes of key taxa in a robust phylogenetic context. High-quality reference genomes not only contribute to true phylogeny inference but also provide a fundamental genomic background for investigating the genetic and molecular basis of phenotypic trait evolution, e.g. modern birds and ruminant animals (Jarvis et al. 2014; Chen et al. 2019). Based on species phylogeny only, bioluminescence appears to have independently emerged at least twice, specifically within Lampyridae–Rhagophthalmidae–Sinopyrophoridae and Elateridae. Further investigation into Luc, which plays a pivotal role in the origin and evolution of bioluminescence (Fallon et al. 2018; Zhang et al. 2020; Al-Handawi et al. 2022), clarified the origin of Luc and bioluminescence. Notably, through the integration of phylogeny, divergence time (Fig. 3a; supplementary figs. S14 and S15 and data S8 and S9, Supplementary Material online), genomic synteny (Fig. 3b; supplementary fig. S16 and data S11 to S12, Supplementary Material online), and biochemical functional analyses (Fig. 3c; supplementary data S10, Supplementary Material online) of beetle Lucs, our study provides compelling evidence for at least 3 parallel origins of bioluminescence, i.e. Lampyridae–Rhagophthalmidae, Sinopyrophoridae, and Elateridae (Fig. 3d). As our study did not include the reference genome of representative species of Phengodidae, we cannot rule out the possibility that bioluminescence also originated independently in Phengodidae and its sister group Rhagophthalmidae due to distant geographies and different morphologies.

Luciferin, the emitter of bioluminescence, is another important component of the bioluminescent system. Previously, we hypothesized a luciferin biosynthesis pathway rooted in the genomic data of luminous beetles from the Lampyridae and Elateridae families (Zhang et al. 2020). In this study, we expanded our investigation beyond Luc to explore the genomic underpinnings of key enzymes related to luciferin, conducted within an enlarged phylogenetic framework that incorporated the genomes of crucial beetle taxa (supplementary note S1 and figs. S21 to S29 and data S13 and S14, Supplementary Material online). Results showed that these enzymes (i.e. phenoloxidase [PO], ATP-binding cassette protein D [ABC-D], peroxisomal membrane protein 2 [Pxmp2], sterol carrier protein X [ScpX], luciferin-regenerating enzyme [LRE], acyl-CoA thioesterase [ACOT], sulfotransferase [ST], and sulfatase [SULF]) exhibited varying copy numbers between luminescent and nonluminescent species (supplementary data S13 and S14, Supplementary Material online). Most enzymes, including PO, Pxmp2, ABC-D, ACOT, SULF, and LRE, retained syntenic one-to-one copies (supplementary figs. S22 to S27, Supplementary Material online). Among these, we previously verified a one-to-one ACOT copy in the firefly *Abscondita terminalis* (*AteACOT1*) that transformed L-luciferin to D-luciferin (Zhang et al. 2020). These findings suggest that the luciferin biosynthesis pathway has undergone a convergent evolutionary process. Concurrently, mutations in Luc, which occurred independently across various beetle family lineages, constitute the key genetic underpinning for the emergence of beetle bioluminescence. At the molecular level, our findings resolve the previously perplexing morphological observation, which troubled Darwin and others: why parallel evolution of bioluminescence occurred in beetles (Darwin 1872; Costa 1975; Branham and Wenzel 2003; Sagegami-Oba et al. 2007; Oba 2009). Parallel evolution is widespread in animals and includes armor plate patterning in wild 3-spine sticklebacks (Colosimo et al. 2005), antifreeze glycoproteins in Antarctic notothenioid fish and Arctic cod (Chen et al. 1997), and poisonous milkweed plant feeding among certain insects such as butterflies, beetles, and aphids (Dobler et al. 2012; Zhen et al. 2012).

We delineated the functional evolution of Luc from its ACS origins. The observed negative trade-offs between luminescence intensity/L-luciferin to D-luciferin transformation and ACS activities in both extant and ancestral Lucs (Fig. 3c) confirmed their dual functionality as both Lucs and ACS enzymes (Oba et al. 2003) and corroborated the “ACS origin” hypothesis that beetle luciferase originated from gene duplication and subsequent neofunctionalization (Khersonsky et al. 2006; Oba et al. 2020). Notably, *EssLuc* (Sinopyrophoridae) exhibited lower luminescence intensity/L-luciferin to D-luciferin transformation ability but the highest ACS activity among the studied Lucs (Fig. 3c). Conversely, its tandem duplicate LL gene (*EssLuc-LL2*) exhibited no luminescence intensity or L-luciferin to D-luciferin transformation ability (Fig. 3c). Considering the later divergence time of *EssLuc* (supplementary fig. S15, Supplementary Material online: 156 Ma) and the lower evolutionary rate of *S. schimmeli* (supplementary fig. S11, Supplementary Material online), it is plausible that the ancestor of *EssLuc* and *EssLuc-LL2* did not emit light, with *EssLuc* potentially undergoing neofunctionalization from its original ACS function to neo-Luc activity.

We verified the pivotal role of LBSs in the evolution of Luc function (Fig. 4; supplementary data S10, Supplementary Material online). Artificially mutating the LBSs in extant Lucs (*LyuLuc1* and *StarLuc*) into a nonluminous pattern resulted in a significant reduction and near disappearance of luminescence intensity and L-luciferin to D-luciferin transformation ability (Fig. 4d and g). In contrast, the activities of LL genes (*AbLL* in click beetle *Agrypnus binodulus*; homologous gene *CG6178* in *Drosophila melanogaster*) showed luminescence activities after the corresponding sites were mutated into the luminous pattern (Oba et al. 2004, 2009; Oba, Iida, et al. 2008; Oba, Shintan, et al. 2008). Of note, although a subclade of Lampyridae LL proteins (*AlaACS1* and *LyuACS1*) possessed the luminous amino acid pattern “TSA,” in vitro experiments showed that they had no luminescence activity (Fig. 3c), explained by their low similarity to Luc. However, *AteACS6*, which belonged to the same subclade as *AlaACS1* and *LyuACS1* (Fig. 3a), emitted faint light (Fig. 3c; supplementary data S10, Supplementary Material online), suggesting that the ancestor of LamLL and AncLamPheRhaLuc may have been bioluminescent, while *AlaACS1* and *LyuACS1* may have lost their luminescence activities.

To further elucidate the evolutionary role of LBSs in Luc function, we performed molecular docking experiments between oxyluciferin and wild-type or mutant Lucs. Among them, *LyuLuc1* and *StarLuc* exhibited high luminescence intensities, with the luminous patterns of “TSA” and “CCA,” respectively. In contrast, *EssLuc* (“LVA”) only showed faint light intensity and was therefore not further analyzed. Within the range of oxyluciferin (3.5 Å), the Luc pockets became more relaxed following mutation (Fig. 4b, c, e, and f). Specifically, the active-site microenvironment (e.g. residues and hydrogen bond) was changed (supplementary table S2, Supplementary Material online), implying that active-site mutations resulted in catalytic activity via changes in the protein structure (DeLuca 1969; Ugarova and Brovko 2002; Bevilaqua et al. 2019). Conformational and active-site microenvironment changes can also affect the color of light emitted by Lucs (Carrasco-Lopez et al. 2018). Thus, we verified the evolutionary trend of Luc bioluminescence spectra, which tended toward hypsochromic shifts and color variations among the different luminous families. Specifically, color divergence occurred during the evolution of Lucs across the different luminous families, including Elateridae (*λ*max = 536 to 592 nm), Sinopyrophoridae (*λ*max = 564 nm), Rhagophthalmidae/Phengodidae (*λ*max = 549 to 622 nm), and Lampyridae (*λ*max = 538 to 583 nm; Fig. 3a; supplementary fig. S19 and data S10, Supplementary Material online). We speculate that complex ecological environments promote complex sequence diversity, as evidenced by the 2 copies of Lucs in fireflies and at least 3 parallel origins of Luc among luminous families. This diversity leads to phenotypic differentiation, including bioluminescent color variation among luminous interfamily and intrafamily and even within individuals (Fig. 3a; supplementary fig. S19 and data S10, Supplementary Material online). For example, Jamaican click beetles (*Pyrophorus plagiophthalamus*; Coleoptera: Elateridae) emit different colored light from ventral and dorsal organs (Stolz et al. 2003).

In summary, genomic and biochemical functional data revealed multiple parallel origins of bioluminescence and functional divergence of Luc within the beetle bioluminescent system. Of note, several gaps between the biochemical functions of these Luc/LL proteins and their organismal functions remain, and further investigations on their functions in organisms are needed to better understand the evolutionary and functional variation mechanisms underlying beetle bioluminescence.

## Materials and Methods

### Sampling and Sequencing

Six beetle species, covering 6 families of Elateroidea, were collected from Southwest China (supplementary fig. S1 and data S1, Supplementary Material online). Some individuals of live adults or larvae from each species were flash-frozen in liquid nitrogen and stored in a −80 °C refrigerator for further use, while other individuals were preserved as voucher specimens in 75% alcohol and deposited at the Kunming Institute of Zoology, Chinese Academy of Sciences, China. Species identification was performed by specialists for each family to ensure accuracy and reliability.

For short-read next-generation sequencing, the whole-genome DNA was extracted from either the whole body or a part of the tissue of a single male/female adult individual or larvae individual using a TIANamp genomic DNA kit (TIANGEN, China) in accordance with the manufacturer's instructions. DNA integrity was checked through agarose gel electrophoresis and quantified using a Nanodrop spectrophotometer and Qubit fluorometer. Paired-end libraries with 350-bp insert size were generated using an NEB Next Ultra DNA Library Prep Kit and sequenced them using Illumina HiSeq4000 sequencers (Novogene, Tianjin, China).

For long-read Nanopore sequencing, genomic DNA was isolated from the same individuals used for next-generation sequencing or another 1 to 2 male adult individuals to construct long DNA fragment libraries following NextOmics’ (Wuhan, China) protocols. Libraries with insertions >20 kb were prepared and sequenced on a PromethION sequencer (Oxford Nanopore, Oxford, UK).

To assist in gene prediction, total RNA was isolated from the whole body/part tissue of either a single male/female adult individual or a larvae individual using TRIzol reagent (Invitrogen, USA) following the manufacturer's instructions. The 350-bp insert size paired-end libraries were generated and sequenced using Illumina HiSeq4000 sequencers (Novogene, Tianjin, China).

### Genome Assembly and Assessment

For Illumina short-reads, the quality per base was evaluated with FastQC (Schmieder and Edwards 2011). Both low-quality reads (>90% bases with quality <Q30) and adaptor sequences were removed. We conducted a 17-mer frequency distribution analysis using Kmerfreq software (Liu et al. 2013). The genome size was calculated using the formula: G = K_num/K_depth, where K_num represents the total number of 17-mers and K_depth represents the expected depth of the analysis.

For Nanopore long-reads, the mean *q*-score par reads <7 were removed. The genome was assembled using WTDBG v2.2 (Ruan and Li 2020) based on clean long reads. To enhance the accuracy of the reference assembly, the primary assembly was polished twice with Pilon v1.12 (Walker et al. 2014) using Illumina short reads. The final assembly was obtained after removing haplotigs and contig overlaps from the polished genome assembly utilizing purge_dups (https://github.com/dfguan/purge_dups). BUSCO (BUSCO v5.2.2; Manni et al. 2021) was used to evaluate the completeness of the genome based on the lineage dataset (insecta_odb10). Illumina short reads were mapped onto the assemblies using BWA v0.7.12 (Li and Durbin 2009) with default settings, and the mapping rate was counted using Samtools v1.13 (Li and Durbin 2009).

### Genome Annotation and Assessment

Before predicting the gene structure, we annotated repeat elements. We used LTR_FINDER v1.05 (Xu and Wang 2007) to discover long terminal repeat retrotransposons and Tandem Repeat Finder v4.07b (Benson 1999) to annotate tandem repeats. We also employed RepeatMasker v4.0.5 (Tarailo-Graovac and Chen 2004) to identify homologous repeats based on the de novo repeat library built by RepeatModeler v1.0.4 (Tarailo-Graovac and Chen 2004), as well as RepeatMasker v4.0.5 (Tarailo-Graovac and Chen 2004) and RepeatProteinMask open-4.0.6 (a package in RepeatMasker with parameters: -no LowSimple -p 0.0001) to search for previously reported repeats in the Repbase library v16.02 (Bao et al. 2015).

Gene structure annotation was conducted through a combination of homology-based prediction, transcriptome-based prediction, and ab initio prediction methods using EvidenceModeler (EVM, v1.1.1; Haas et al. 2008). For homology-based predictions, we aligned protein data sets of 5 beetles (supplementary data S1, Supplementary Material online) to the repeats soft-masked genome by TBLASTN (Gertz et al. 2006), with an E-value <1e−5. Identified homologous sequences were then subjected to GeneWise v2.2.0 (Birney and Durbin 2000) to define gene models. For transcriptome-based prediction, we employed HISAT v2.2.1 and StringTie v2.1.6 (Pertea et al. 2016) to assemble transcriptomes. Transcript sequences were then mapped to corresponding assembled genomes using the Program to Assemble Spliced Alignments (PASA; Haas et al. 2003) to generate a full-length cDNA set that was used to train the ab initio gene prediction programs. The AUGUSTUS v3.4.0 (Hoff and Stanke 2013) was executed to predict coding regions in the repeat-masked beetle genomes. Finally, these gene models in combination with the assembled transcriptome were integrated by combining all pieces of evidence into a nonredundant consensus set of genes using EVM. The consensus gene set was further updated using 2 rounds of PASA to obtain improved gene structure.

To evaluate the quality of predicted genes, we compared various gene features such as mRNA length, CDS length, exon length, intron length, and exon number between our assembled genomes in this study and those published genomes of 4 luminous beetle species, namely *Lamprigera yunnana*, *A. terminalis*, *P. pyralis*, and *I. luminosus*. Furthermore, we employed BUSCO v5.2.2 (Manni et al. 2021) to evaluate the completeness of annotated genes.

Gene function was predicted using BLASTP searches (*E*-value <1e−05) against various databases, including SwissProt, TrEMBL, and NCBI nonredundant (NR) proteins database. The structural domains and motifs of all genes were scanned against SMART, ProDom, Pfam, PRINTS, PROSITE, and PANTHER databases using InterProScan v5.25 (Jones et al. 2014). The Gene Ontology (GO) terms were extracted based on the corresponding InterPro entry. The metabolic pathways in which the genes might be involved were assigned by BLASTP searches (E-value <1e−05) against the Kyoto Encyclopedia of Genes and Genomes (KEGG) protein database (Kanehisa and Goto 2000).

### Phylogenomic Data Matrix Construction and Phylogenomic Analyses

We generated 6 data matrices (supplementary data S1, Supplementary Material online) to investigate the phylogenetic relationship among the luminous beetle lineage.

#### Data Matrix #1 to #3—Orthologous Genes

Combined with the genomes assembled in this study and published, 15 beetle genomes containing gene annotation information can be used. The protein sets were clustered into families by running reciprocal BLAST analysis in OrthoFinder v2.4.0 (Emms and Kelly 2019). Five hundred sixty-eight single-copy orthologous genes were identified. Each gene was aligned using MAFFT v7.487 (Katoh et al. 2019) with default parameters at the amino acid level and nucleotide level, respectively. Ambiguously aligned regions were removed using trimAl v1.4.rev22 (Capella-Gutierrez et al. 2009) with parameter: “-gt 0.5.” The remaining alignments were then concatenated into data matrix #1 (AA: 348,711 amino acid sites), data matrix #2 (codon: 1,046,133 nucleotide sites), and data matrix #3 (codon12: 592,961 nucleotide sites).

#### Data Matrix #4—BUSCO Genes With 15 Taxa

We extracted the single-copy protein BUSCO genes of 15 beetles that correspond to data matrices of orthologous genes. The alignments of these 992 BUSCO genes, each of which has >80% taxon occupancy, were then concatenated into data matrix #2 with 450,247 amino acid sites.

#### Data Matrix #5—BUSCO Genes With 19 Taxa

To lessen the potential effects of limited sampling, we added the single-copy genome BUSCO genes from 4 taxa (Cantharidae) and obtained 949 single-copy BUSCO genes. A data matrix with 19 taxa and 429,703 amino acid sites was produced after alignment and filtration.

#### Data Matrix #6—WGAs

After strict quality control, we selected 11 high-quality reference genomes for multiple WGAs that were performed using Cactus v1.0.0 (Armstrong et al. 2020). Then, the MAF-format alignments were obtained using hal2maf (–refGenome Ppy –onlyOrthologs –noAncestors –hdf5InMemory). The poorly aligned regions were filtered by trimAl v1.4.rev22 (Capella-Gutierrez et al. 2009) with parameter: “-gt 0.8.” This data matrix contains 26,771,266 nucleotide sites.

For the 6 data matrices constructed above, concatenation-based and coalescent-based approaches were used to infer the phylogenetic relationship among the luminous beetle lineage. All phylogenetic analyses were performed using IQ-TREE v2.1.3 (Minh et al. 2020) based on the maximum likelihood (ML) method.

#### Concatenation-Based Approach

For concatenation-based analyses, “HKY/TIM3+F+R6” and “Q.insect/LG+F+R5” models of substitution were used for each data matrix at the nucleotide and amino acid levels, respectively. Two independent runs were employed in all data matrices, and the topological robustness was evaluated by 1,000 ultrafast bootstrap replicates (Hoang et al. 2018).

#### Coalescent-Based Approach

Individual gene trees were inferred using IQ-TREE v2.1.3 with an insect model (Q.insect+F+R5) at the amino acid level and automatic detection for the best-fitting model with “-MFP” option using ModelFinder (Kalyaanamoorthy et al. 2017) under the Bayesian information criterion at the nucleotide level. For each gene tree, we conducted 2 independent tree searches to obtain the best-scoring ML tree with the “–runs 2” option. The topological robustness of each gene tree was evaluated by 1,000 ultrafast bootstrap replicates. We used the individual ML gene trees to infer the coalescent-based species tree using ASTRAL-III v5.6.3 (Zhang, Che, et al. 2018; Zhang, Rabiee, et al. 2018) for each data matrix. The topological robustness was evaluated using the local posterior probability.

#### Analyses of Gene Tree Discordance and Alternative Relationships

DiscoVista v1.0 (Sayyari et al. 2018) was used to quantify and visualize gene tree discordance for alternative topologies in the orthologous genes and BUSCO genes. We also applied FcLM analysis (Strimmer and von Haeseler 1997; Misof et al. 2013) to investigate potential incongruent or confounding signals among the Cantharidae and Sinopyrophoridae as described by Kusy et al (2021). The tree-likeness graph for the 3 possible quartet topologies shows the support for each topology. For Cantharidae, we binned sequenced species into 4 clusters: (i) Cantharidae, (ii) Elateridae, (iii) Lampyridae + Rhagophthalmidae + Sinopyrophoridae, and (iv) Lycidae + Buprestidae + Tenebrionidae + Curculionidae + Scarabaeidae + Silphidae. For Sinopyrophoridae, we binned sequenced species into 4 clusters: (i) Sinopyrophoridae, (ii) Rhagophthalmidae + Lampyridae, (iii) Elateridae, and (iv) Cantharidae + Lycidae + Buprestidae + Tenebrionidae + Curculionidae + Scarabaeidae + Silphidae. FcLM analysis was conducted using TREE-PUZZLE v5.3.rc16 (Schmidt et al. 2002), applying the “LG” substitution matrix on amino acid sites or the “HKY” substitution matrix on nucleotide sites.

### Comparative Genomic Analyses

To detect the evolutionary features of the luminous beetle lineages, we used 10 intra-Elateroidea and 6 extra-Elateroidea for comparative genomic analysis (supplementary data S1, Supplementary Material online).

#### Gene Family Expansion/Contraction

To evaluate gene family expansion and contraction in the luminous beetle lineage, we used the OrthoMCL v2.0.9 (Li et al. 2003) method on the all-versus-all BLASTP alignments to construct gene families. Then, CAFÉ v3.1 (De Bie et al. 2006) with the ML method across a user-specified divergence time tree was used to analyze the expansion and contraction of gene families. If the number of genes in one gene family was >100, it was filtered. If the *P*-value was <0.01, the gene family would be taken as having undergone significant expansion or contraction. In addition, GO and KEGG enrichments of genes in expanded or contracted families were carried out using the clusterProfiler package (Yu et al. 2012) in R software with a *P*-value < 0.05.

#### Positive Selection and Rapid Evolution Analyses

All 568 single-copy orthologous genes were generated by running reciprocal BLAST analysis in OrthoFinder v2.4.0 (Emms and Kelly 2019). Each ortholog was aligned using PRANK v.170427 (Loytynoja and Goldman 2005) and filtered gaps by GBlocks v0.91b (Castresana 2000) with the parameter “-t = c -b4 = 5.” We estimated the values of *Ka*, *Ks*, and *ω* (*Ka*/*Ks*) for the concatenated alignment of these orthologs using the codeml program of PAML v4.9 (Yang 2007) with the free ratio model for each branch. To estimate the lineage-specific evolutionary rate, the average *Ka*/*Ks* of each species was estimated using 10,000 concatenated alignments constructed from 150 randomly chosen orthologs.

We used the optimized branch-site model to detect PSGs following the author's recommendation (Zhang et al. 2005). The significance of differences compared with the null model was evaluated using likelihood ratio tests (LRTs) by calculating twice the loglikelihood of the difference following a χ^2^ distribution. Genes with *P*-values <0.05 were identified as candidates that underwent positive selection.

Also, we identified REGs at the same branches as PSGs. The branch model in PAML was used, with the null model assuming that all branches have been evolving at the same rate and the alternative model allowing the foreground branch to evolve at a different rate. The LRT with df = 1 was used to discriminate between alternative models for each ortholog in the gene set. Genes with *P-*value < 0.05 and a higher *ω* value for the foreground than the background branches were considered evolving at a significantly faster rate. In addition, GO and KEGG enrichments of PSGs and REGs were carried out using the clusterProfiler package (Yu et al. 2012) in R software with *P*-value < 0.05.

### Genomic Context of Luc Evolution

Lucs are the key enzymes that catalyze the light-emitting reactions in bioluminescence and belong to the ACS superfamily (Viviani 2002). Based on the specificity such as chain length of the carboxylate substrate, the ACS superfamily can be classified into 9 families: Luc and LL family (LLL), 4-coumarate: CoA ligase family (4CL), ACS short-chain family (ACSS), ACS medium-chain family (ACSM), ACS bubblegum family (ACSBG), very long-chain ACS (ACSVL). Members of this family were also investigated as fatty acid transport proteins (FATP), ACS long-chain family (ACSL) and other families (ACS family member 2 (ACSF2); ACS family member 3 (ACSF3); aminoadipate-semialdehyde dehydrogenase (AASDH = ACSF4; Khurana et al. 2010; Watkins and Ellis 2012).

#### Identification of Luc and ACS Superfamily

Luc and ACS of the assembled genomes were identified according to the previous study (Zhang et al. 2020). Briefly, a hmm profile was built by HMMER v3.3 (Finn et al. 2015) based on the ACS encoding genes from *D. melanogaster* and *Homo sapiens* and then used as a reference in hmmsearch to identify all candidate ACS genes. The protein sequences of all ACS genes were aligned using MAFFT v7.487 (Katoh et al. 2019) with default parameters. The poorly aligned regions were trimmed using trimAl v1.4.rev22 (Capella-Gutierrez et al. 2009; gt = 0.5). FastTree 2 (Price et al. 2010) was used to build the gene tree of the candidate ACS genes for classification. We also combined the LLL clade, 4CL clade, cloned beetle Luc genes, and other cloned Luc paralogs (supplementary data S10, Supplementary Material online) to further construct the phylogenetic tree.

#### Clade Stability Analyses

MIPhy was used to identify clades (4CL and LLL) and assess their stability (Curran et al. 2018). We selected 7 species, representing lineages of 4 luminous families (Lampyridae, Rhagophthalmidae, Sinopyrophoridae, and Elateridae) and 2 adjacent nonluminous families (Lycidae and Cantharidae). We first extracted 4CL and LLL genes from each species and aligned the sequences using MAFFT v7.487 (Katoh et al. 2019) with default parameters. The poorly aligned regions were removed using trimAl v1.4.rev22 (Capella-Gutierrez et al. 2009; gt = 0.5). A phylogenetic tree was constructed using IQ-TREE v2.1.3 with the ModelFinder function to determine the best-fit model (Minh et al. 2020). We rooted the tree using the midpoint.root function in the FigTree v1.4.4 (http://tree.bio.ed.ac.uk/software/figtree/). The MIPhy online version (http://miphy.wasmuthlab.org/) was used to quantify phylogenetic instability with default parameter settings. We also estimated *Ka*/*Ks* ( $\underline{\omega }$) based on the numbers of synonymous and nonsynonymous substitutions for the branch of one LLL subclade (including Luc genes) using the codeml program of PAML v4.9 (Yang 2007) under the free ratio model (model = 1, NS sites = 0). GeneRax v2.0.4 (Morel et al. 2020) was employed to reconcile gene family trees with species trees.

#### Phylogeny and Evolution of Luc and Other LL Genes

The phylogenetic tree of all LLL genes was constructed using FastTree 2 (Price et al. 2010). DmeACS (4CL, *CG9009*) was used as an outgroup. MCMCtree program in PAML v4.9 (Yang 2007) was used to estimate the divergence time of LLL genes with the approximate likelihood calculation method. The MCMC convergence was checked using Tracer v1.7.2 (Rambaut et al. 2018) and confirmed with 2 independent runs. The tree files were visualized using FigTree v1.4.4 (http://tree.bio.ed.ac.uk/software/figtree). We further checked the phylogenetic relationship of LLL in the luminous branch using IQ-TREE v2.1.3 (Minh et al. 2020) with settings “–runs 2 -B 1000 –boot-trees -m Q.insect + G4.” The ML tree was visualized and modified in iTOL v6.5 (Letunic and Bork 2021).

#### Gene Synteny and Collinearity of Luc Genes

To explore the homologous systemic blocks, we checked the conserved syntenic blocks surrounding the Luc locus across genomes of 7 luminous beetles (Lampyridae: *L. yunnana*, *A. terminalis*, *V. saturnalis*, and *P. pyralis*; Rhagophthalmidae: *M. giganteus*; Sinopyrophoridae: *S. schimmeli*; and Elateridae: *I. luminosus*). Protein similarity was obtained based on all-to-all Blastp (-evalue 1e-10, -num_alignments 20). Per block containing >3 homologous gene pairs was regarded as conserved collinear blocks, in which the identity of gene pairs from different species was >50% and the coverage is >80%. The syntenic relationships between 7 luminous species were visualized using MCScan (https://github.com/tanghaibao/jcvi/). WebLogo v3 (http://weblogo.threeplusone.com/; Crooks et al. 2004) was used to generate of sequence logos.

### Protein Structure Analyses

#### Ancestral Luc Sequence Prediction

The phylogenetic tree of LLL in the luminous branch and related alignments were applied to the codeml program of PAML v4.9 (Yang 2007) to infer 6 ancestral Luc sequences, with the empirical model of Jones matrix, including that of Lampryidae *Luc1* (AncLamLuc1), that of Lampyridae *Luc2* (AncLamLuc2), that of Lampyridae *Luc1* and *Luc2* (AncLamLuc), that of Phengodidae–Rhagophthalmidae Luc (AncPheRhaLuc), that of Lampyridae-Phengodidae–Rhagophthalmidae Luc (AncLamPheRhaLuc), and that of Sinopyrophoridae (AncSinLuc; Fig. 3a; supplementary table S3, Supplementary Material online). The average posterior probability of the amino acid residues in the 6 reconstructed ancestral Lucs was sufficiently high, ranging from 0.91 to 0.97 (supplementary table S3, Supplementary Material online). Although posterior probabilities of several sites were low, those of most sites including LBSs of Luc (Sites 346 to 348) were extremely stable (supplementary data S15, Supplementary Material online).

#### Homology Modeling and Molecular Docking

The Luc protein 3D structure was predicted with the AlphaFold-Colab (no templates; https://colab.research.google.com/github/sokrypton/ColabFold/blob/main/AlphaFold2.ipynb; Mirdita et al. 2022) using the default setting. The best structure was chosen based on the pLDDT (predicted local distance difference test; a per-residue confidence metric) and PAE (predicted aligned error) scores. The structure of oxyluciferin (2-(6-HYDROXY-1,3-BENZOTHIAZOL-2-YL)-1,3-THIAZOL-4(5H)-ONE) was downloaded from Protein Data Bank in Europe (https://www.ebi.ac.uk/pdbe/entry/pdb/2d1r/bound/OLU) for docking. Protein preparation was done by removing water molecules from the structure and adding hydrogen using Autodock v4.2.6 software (Harris et al. 2008). The contact energies grid maps of various kinds of atoms were precalculated using AutoGrid 4.2. The grid box covering the whole protein size was built in each dock. Flexible ligand docking into a rigid protein environment was conducted with chosen parameters using AutoDock 4.2: the number of GA runs was 10; the maximum number of generations was 250,000; the gene mutation rate was 0.02 to define and test the relationship between oxyluciferin and Luc. Default settings were used for all other parameters. The docking result was validated by measuring the RMSD and visualized using PyMol v2.5.2 (Schrödinger 2021). PyMol was used to compare the protein structures of Lucs.

### Characteristics of Lucs and Their Ancestor Proteins, and LL Proteins

#### Molecular Cloning of Lucs and Their Ancestor Proteins, and LL Proteins

Total RNA was isolated from the whole adult of *S. schimmeli* using Trizol (Life Technologies, CA) and then reverse transcribed into double-strand cDNA using the PrimeScript RT-PCR Kit (Takara, Japan). The double-strand cDNA was maintained at −20 °C. We amplified the Luc and LL genes of *S. schimmeli* (*EssLuc* and *EssLuc-LL2*) based on the primers (supplementary table S4, Supplementary Material online). The PCR conditions were set as follows: 98 °C, 30 s; 35 cycles (98 °C, 10 s; 58 °C, 10 s; 72 °C, 1 min); 72 °C, 2 min. Ancestral Luc sequences (AncLamLuc1, AncLamLuc2, AncLamLuc, AncPheRhaLuc, AncLamPheRhaLuc, and AncElaLuc), extant Luc (*VesLuc1*, *VesLuc2*, and *StarLuc*) and LL sequences (*AlaACS1*, *AteACS6*, *LyuACS1*, *SperPACS3*, and *EssPACS2*), and site-directed mutants (*LyuLuc1_M* and *StarLuc_M*) were optimized and synthesized by the company of Genecreate (Wuhan, China), except *EssLuc* and *EssLuc-LL2* (*S. schimmeli*) that are cloned in this study and *LyuLuc1* (*L. yunnana*) that was cloned in our previous work (Liu et al. 2019).

#### Expression and Purification of Recombinant Proteins

The methods to culture and extract the recombinant LLL proteins followed our previous works (Liu et al. 2019; He et al. 2021). Briefly, the amplified products (*EssLuc* and *EssLuc-LL2*) were inserted into pEASY-T5 (Transgen) to produce the plasmids, from which the gene fragments were digested with *NdeI* and *XhoI* enzymes and then inserted into pCold-TF (Takara, Japan) to obtain the expression plasmid. Other genes synthesized by the company of Genecreate (Wuhan, China) were cloned into a pCold-I plasmid for expression. These expression plasmids were transformed into *Escherichia coli* BL21 (DE3; Tsingke, China) and expressed at 15 °C. Then, the proteins were purified using a nickel-nitrilotriacetic acid (Ni-NTA) column (Qiagen, Germany). The obtained proteins were stored at −80 °C, and the concentration was measured using an Enhanced BCA Protein Assay Kit (Beyotime, China).

#### Measurement of the Luminescence Intensity and Spectra

Luminescence intensities described in relative light units were measured using a luminescence Octa AB-2270 (ATTO, Tokyo, Japan) for 20 s. A total of 10 µL 0.1 mg/mL purified recombinant protein solution was mixed with 50 µL substrate solution (2 mM ATP, 0.5 mM D-luciferin, and 4 mM MgCl_2_ in 100 Mm Tris-HCl buffer with pH 8.0) for measuring luminescence intensity. Luminescence spectra were measured using a spectrophotometer AB-1850 (ATTO, Tokyo, Japan) under 1 min exposure time. The spectral sensitivity was calibrated according to the method of publication (Oba et al. 2020). One microgram of purified recombinant Luc was mixed with 0.5 mM D-luciferin, 4 mM ATP, and 4 mM MgCl_2_ in 0.1 M Tris-HCl (pH 8.0) for measuring spectra. For proteins with weak luminescence intensity, 5 μg or more recombinant protein was used for measuring their spectra. All assays including measurement of the luminescence intensity and spectra here, and measurements of L-luciferin to D-luciferin transformation ability and of ACS activity in the succeeding parts were performed in triplicate. *LyuLuc1*, one Luc cloned in our previous work (Liu et al 2019), was used as positive control, and ddH_2_O as negative control.

#### Measurement of L- to D-Luciferin Transformation Ability

Our previous work confirmed the acyl-CoA thioesterase 1 of *A. terminalis* (*AteACOT1*), together with luciferase, can promote the high-efficiency inversion of L-luciferin into D-luciferin (Zhang et al 2020). The activity of converting L-luciferin to D-luciferin for Lucs was measured by the coupling luminescence intensity of the reaction mixture. The reaction mixture (200 μL) for this measurement contained 0.1 mM L-luciferin, 8 mM MgSO_4_, 3 mM ATP, 0.5 mM COASH, 1 μg AteACOT1, and 1 μg recombinant proteins in 100 mM Tris-HCl (pH = 8.0). The reaction mixture was incubated at 30 °C for 45 min and then detected using Octa AB-2270 (ATTO, Tokyo, Japan) for 20 s.

#### Measurement of the ACS Activity

Following the reported method (Oba et al. 2020), we measured the initial rate of AMP formation by coupling the thioesterification reaction with adenylate kinase, pyruvate kinase, and lactate dehydrogenase and monitoring the oxidation of the reduced form of nicotinamide adenine dinucleotide (NADH) at 340 nm (6220 M^−1^ cm^−1^; Kato et al. 2013). The reaction solution (total volume was brought to 100 μL) for this measure containing 100 mM Tris-HCl (pH 8.0), 10 mM ATP, 10 mM MgCl_2_, 0.35 mM lauric acid (C12:0) substrate, 2 mM CoASH, 1 mM phosphoenolpyruvic acid, 0.4 mM NADH, adenylate kinase (40 μg/mL), pyruvate kinase (20 μg/mL), and lactate dehydrogenase (20 μg/mL) was incubated at room temperature (27 ± 2 °C) for 10 min, and 2 μg of purified recombinant protein was added for measurement. The data were collected at 5 s intervals for 10 min reaction using Multiskan GO (Thermo Scientific, Germany) operated with professional software (SkanIt RE v6.1).

## Supplementary Material

msad287_Supplementary_Data

## Data Availability

All data on the 6 beetles were submitted to NCBI (BioProject: PRJNA884806). Illumina short reads, Nanopore reads, and RNA reads were submitted to SRA with accession numbers: SRR22077043 to SRR22077061 and SRR12206127 (BioProject: PRJNA645732). Genome assemblies are available at NCBI with accession numbers: JARFJ[A|B|W|X|Y|Z]000000000 and in the Science Data Bank (https://cstr.cn/31253.11.sciencedb.07158).
