# Supplementary material for: Multiple Origins of Bioluminescence in Beetles and Evolution of Luciferase Function
Source: Mol Biol Evol. 2024 Jan 4;41(1):msad287. doi: 10.1093/molbev/msad287 (PMC10798137; doi:10.1093/molbev/msad287)
Supplement: msad287_Supplementary_Data [file msad287_supplementary_data.zip › MBE-23-0208_final_R3_Supplementary Information_20231215.pdf]

Supplementary note 1. Genes related to the pathway of luciferin metabolism

1 Key enzymes in the pathway of luciferin metabolism.

Bioluminescent beetles produce light within peroxisomes of photocytes located in morphologically diverse luminous organs (Buck 1948) via a shared luciferase-luciferin system (Viviani 2002). Luciferin (D-2-(6-hydroxy-2-benzothiazolyl)-2-thiazoline-4-carboxylic acid), the true emitter of bioluminescence, is an important component of the bioluminescent system. How luciferin is biosynthesized and metabolized in luminous beetles has long been a very important topic in bioluminescence research. Previously, we hypothesized a luciferin biosynthesis pathway rooted in the genomic data of luminous beetles from the Lampyridae and Elateridae families (Zhang et al. 2020). In this study, we expanded our investigation beyond luciferase to explore the genomic underpinnings of key enzymes related to luciferin, conducted within an enlarged phylogenetic framework that incorporated the genomes of crucial beetle taxa, including genomes of luminescent species (*Vesta saturnalis*, *Menghuoius giganteus*, and *Sinopyrophorus schimmeli*) and closely related non-luminescent species (*Sinelater perroti*, *Lycocerus yunnanus*, and *Platerodrilus igneus*). Enzymes include phenoloxidase (PO), peroxisomal membrane protein 2 (Pxmp2), ATP-binding cassette protein D (ABC-D), sterol carrier protein X (ScpX), acyl-CoA thioesterase (ACOT), sulfotransferase (ST) and luciferin sulfotransferase (LST), sulfatase (SULF), and luciferin-regenerating enzyme (LRE).

2 Gene identification and classification

The full method of enzyme-coding gene identification and classification is completely based on our previous article (Zhang et al. 2020). Briefly, we used the homologous protein sequences of fruit files and fireflies as references. The systematic BLASTP (E-value <1e-5) search key enzymes of beetle genomes was performed using references as queries, except Pxmp2 which searched the domain PF04117 (Mpv17/PMP22 family). After removing redundancy, top hits for putative genes were

retained. Candidate genes were further verified using BLASTP versus non-redundant NCBI protein sequences (NR database). Protein sequences of each gene were aligned using MAFFT v7.487 (Katoh et al. 2019) with default parameters. Poorly aligned regions were trimmed using trimAl v1.4.rev22 (Capella-Gutierrez et al. 2009) (gt = 0.5). For classification, a phylogenetic tree was constructed using IQ-TREE v2.1.3 with the ModelFinder function to determine the best-fit model (Minh et al. 2020). Gene trees were visualized and modified using FigTree v1.4.4 (http://tree.bio.ed.ac.uk/software/figtree/) or iTOL v6.5 (Letunic and Bork 2021).

3 Gene synteny and collinearity

To explore homologous systemic blocks, we firstly check if there are one-to-one gene copies between different species (Lampyridae: *Lamprigera yunnana*, *A. terminalis*, *V. saturnalis*, and *P. pyralis*; Rhagophthalmidae: *M. giganteus*; Sinopyrophoridae: *S. schimmeli*; Elateridae: *Ignelater luminosus*, *S. perroti*; Cantharidae: *L. yunnanus*; Lycidae: *P. igneus*), and if so, perform collinearity analysis. Secondly, we checked conserved syntenic blocks surrounding the gene locus across genomes. Protein similarity was obtained based on all-to-all Blastp (-evalue 1e-10, -num_alignments 20). Per block containing more than three homologous gene pairs was regarded as conserved collinear blocks, in which the identity of gene pairs from different species was more than 50% and the coverage is greater than 80%. Thirdly, syntenic relationships between seven luminous species were visualized using MCScan (https://github.com/tanghaibao/jcvi/).

References

Buck JB. 1948. The anatomy and physiology of the light organ in fireflies. *Annals of the New York Academy of Sciences* 49:397–485.

Capella-Gutierrez S, Silla-Martinez JM, Gabaldon T. 2009. trimAl: a tool for automated alignment trimming in large-scale phylogenetic analyses. *Bioinformatics* 25:1972–1973.

Katoh K, Rozewicki J, Yamada KD. 2019. MAFFT online service: multiple sequence alignment, interactive sequence choice and visualization. *Briefings in Bioinformatics* 20:1160–1166.

Letunic I, Bork P. 2021. Interactive Tree Of Life (iTOL) v5: an online tool for phylogenetic tree display and annotation. *Nucleic Acids Research* 49:W293–W296.

Minh BQ, Schmidt HA, Chernomor O, Schrempf D, Woodhams MD, von Haeseler A, Lanfear R. 2020. IQ-TREE 2: New models and efficient methods for phylogenetic inference in the genomic era. *Molecular Biology and Evolution* 37:1530–1534.

Viviani VR. 2002. The origin, diversity, and structure function relationships of insect luciferases. *Cellular and Molecular Life Sciences* 59:1833–1850.

Zhang R, He JW, Dong ZW, Liu GC, Yin Y, Zhang XY, Li Q, Ren YD, Yang YZ, Liu W, et al. 2020. Genomic and experimental data provide new insights into luciferin biosynthesis and bioluminescence evolution in fireflies. *Scientific Reports* 10:1–19.

Supplementary Figures:

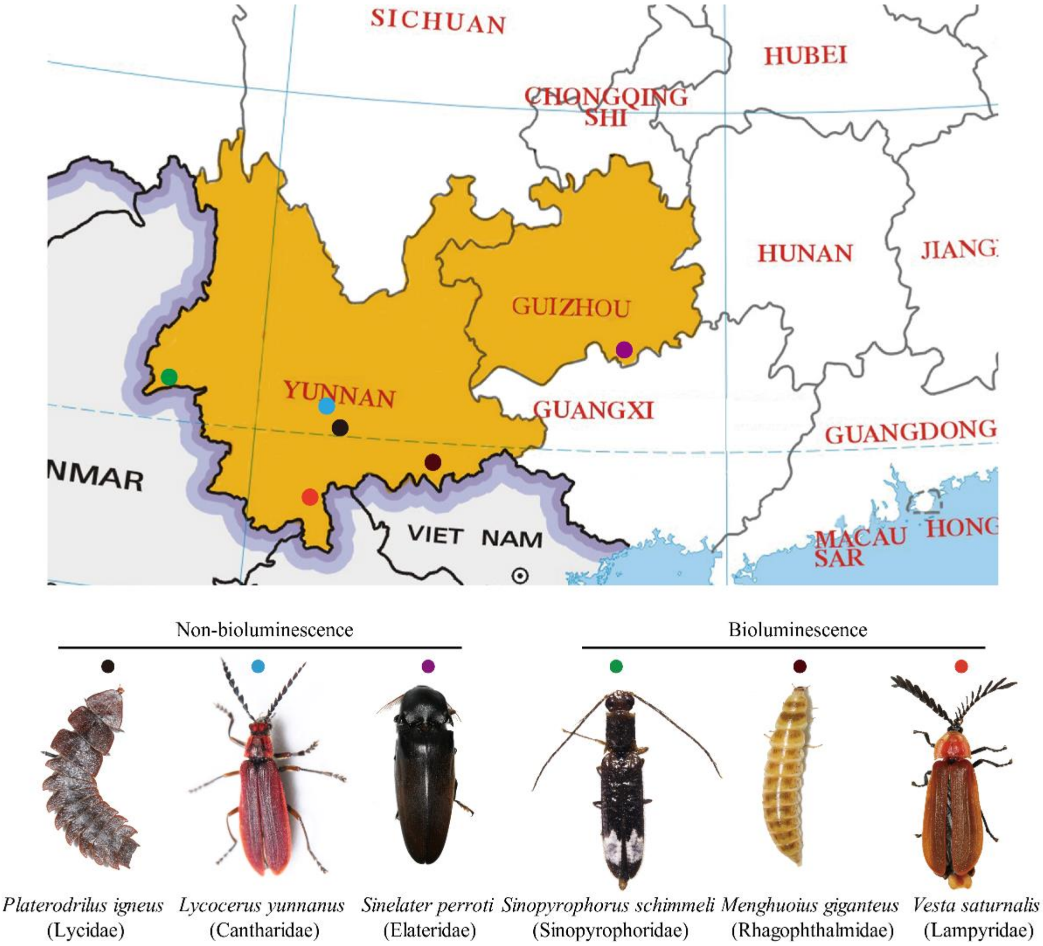

Supplementary fig. 1. Collection sites of sequenced species in this study.

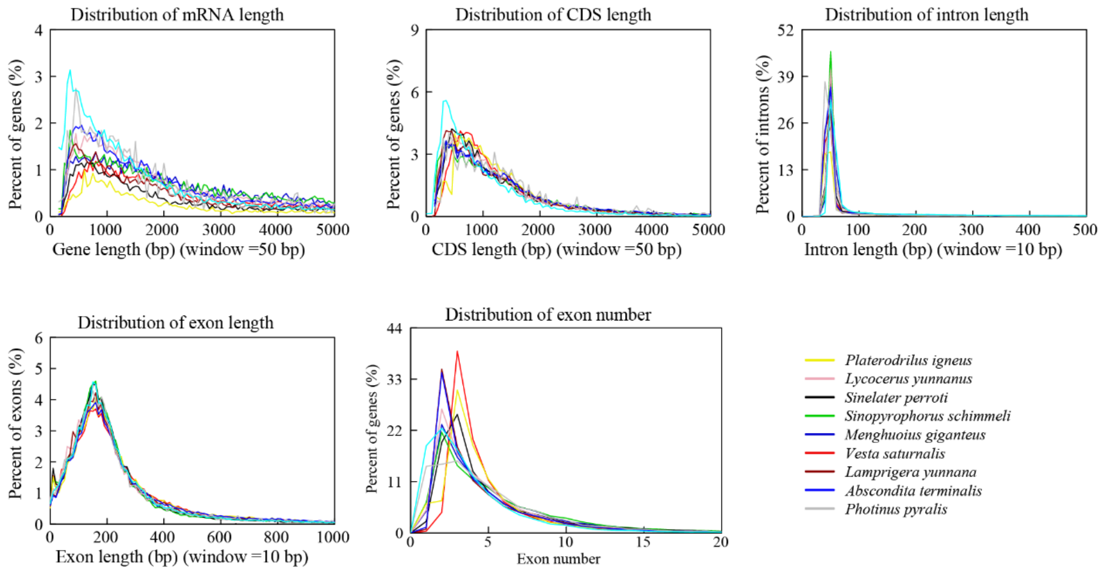

Supplementary fig. 2. Comparison of gene features among genomes of nine Elateroidea beetle species, including six sequenced in this study (*Platerodrilus igneus*, *Lycocerus yunnanus*, *Sinelater perroti*, *Sinopyrophorus schimmeli*, *Menghuoius giganteus*, *Vesta saturnalis*) and three firefly species sequenced previously (*Photinus pyralis*, *Abscondita terminalis*, *Lamprigera yunnana*). CDS: protein-coding sequence.

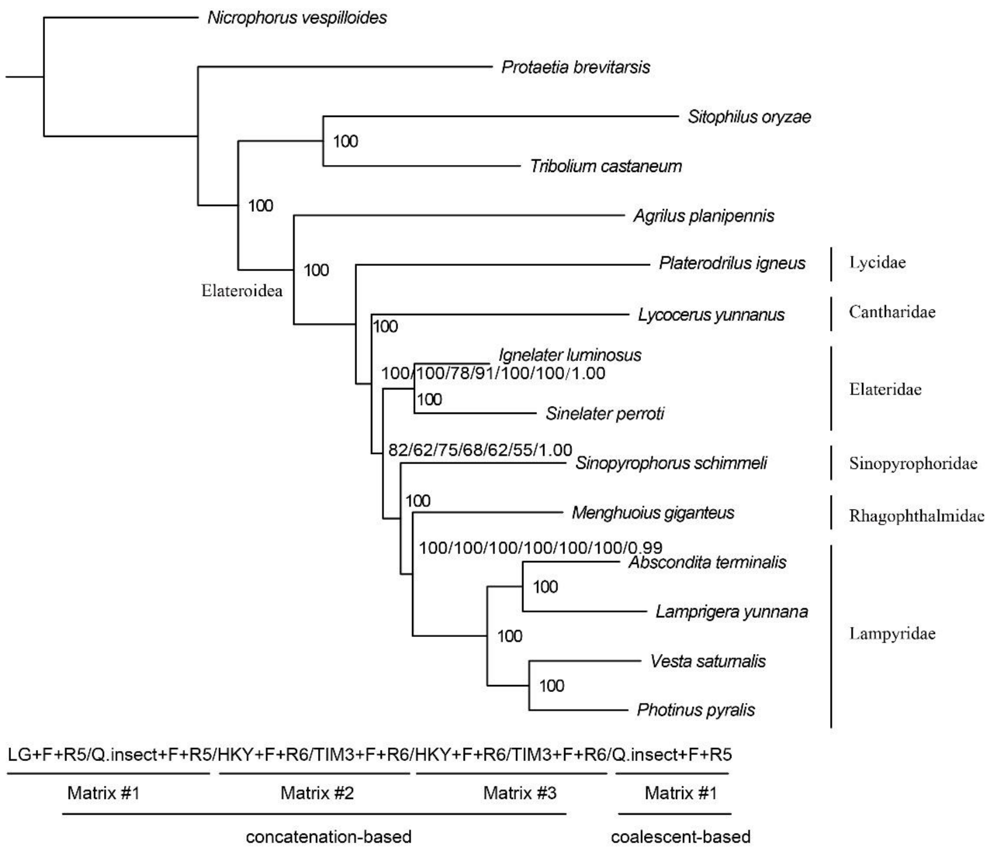

Supplementary fig. 3. Phylogenetic tree based on three data matrices: data matrix #1 (DNA: 348,711 amino acid sites), data matrix #2 (codon: 1,046,133 nucleotide sites), and data matrix #3 (codon12: 592,961 nucleotide sites) derived from 568 single-copy orthologous genes among 15 taxa. Concatenation-based and coalescent-based approaches were used in iqtree2 and ASTRAL-III, respectively. The ultrafast bootstrap with 1000 replicates and local posterior probability are labeled on each node. The presence of a single number 100 across different data matrices indicates the ultrafast bootstrap or posterior probability in each node is 100 or 1.00.

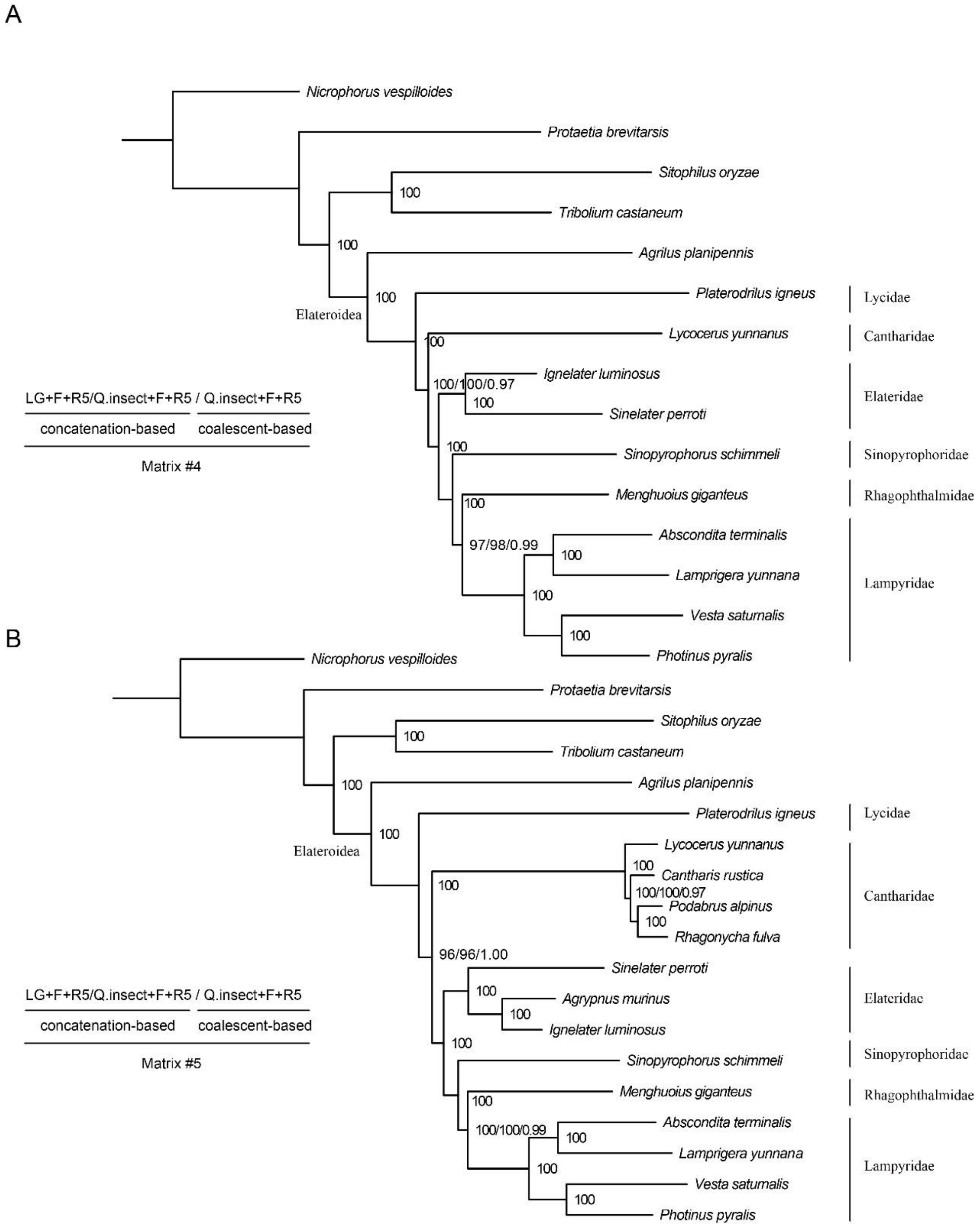

Supplementary fig. 4. Phylogenetic tree based on single-copy BUSCO genes. Concatenation-based and coalescent-based approaches were used in iqtree2 and ASTRAL-III, respectively. The ultrafast bootstrap with 1000 replicates and the local posterior probability are labeled on each node. The presence of a single number 100 across different data matrices indicates the ultrafast bootstrap or posterior probability in each node is 100 or 1.00. A. Phylogenetic tree based on the data matrix #4 (AA: 450,247 amino acid sites) that derived from 992 single-copy protein BUSCO genes

among 15 taxa. B. Phylogenetic tree based on the data matrix #5 (AA: 429,703 amino acid sites) that derived from 949 single-copy BUSCO genes among 15 taxa (pep BUSCO) and 4 taxa (*Cantharis rustica*, *Podabrus alpinus*, *Rhagonycha fulva*, and *Agrypnus murinus*) (genome BUSCO).

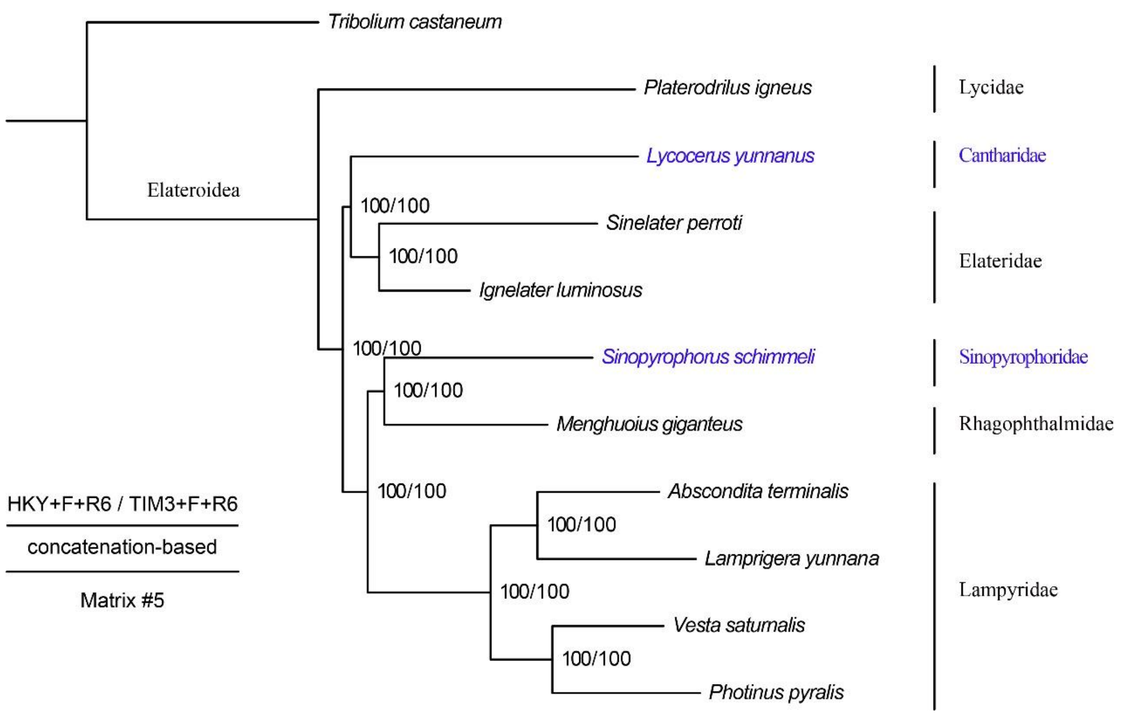

Supplementary fig. 5. Whole-genome alignment tree based on the data matrix #6 (26,771,266 nucleotide sites) that derived from high-quality reference genomes among 11 taxa, with concatenation-based approach in iqtree2 (--run 2 -m HKY+F+R6/TIM3+F+R6). The ultrafast bootstrap with 1000 replicates is labeled on each node. Discordant species with Fig. 1 displayed with deep blue.

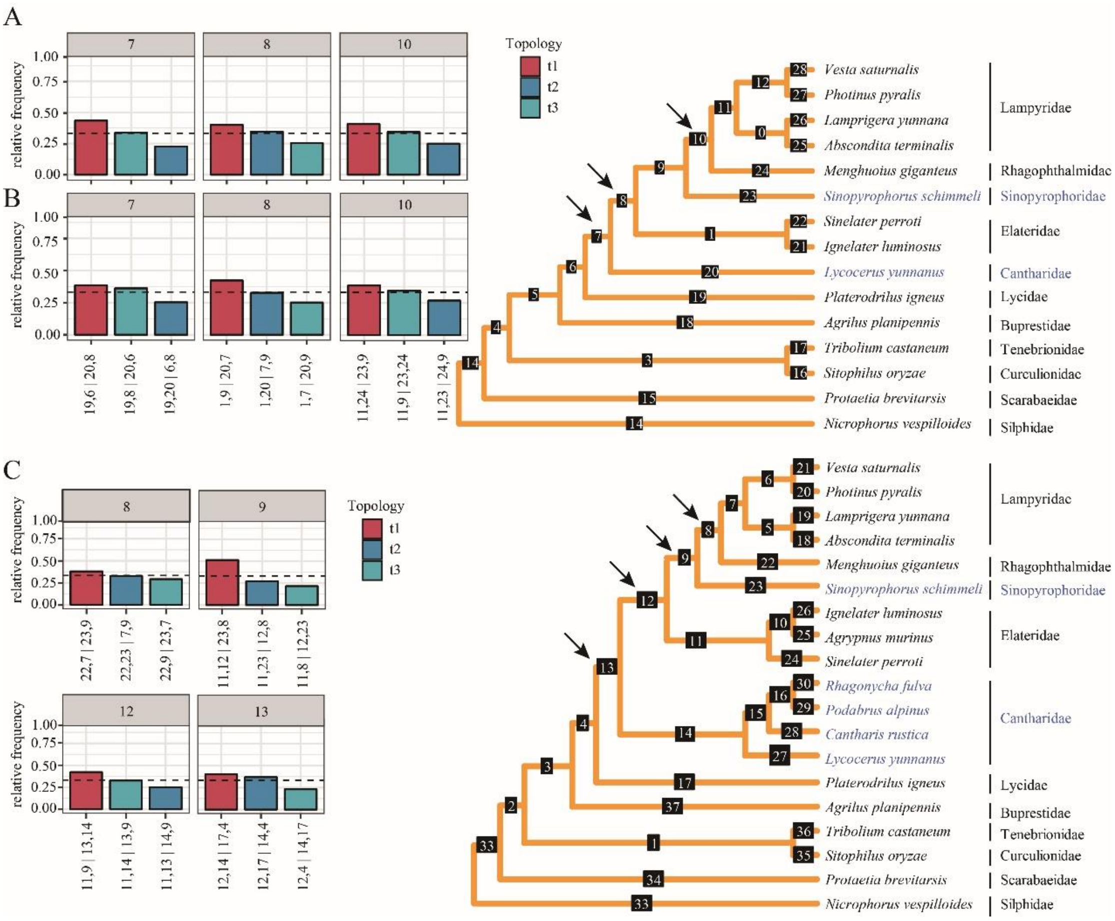

Supplementary fig. 6. DiscoVista relative frequency analyses of three topologies (t1–t3) generated from 568 single-copy orthologous genes among 15 taxa (A), 992 single-copy BUSCO genes among 15 taxa (B), and 949 single-copy BUSCO genes among 19 taxa (C). Each gene tree was constructed using iqtree2 (--run2 -m Q.insect+F+R5). Discordant species are displayed in deep blue. Each internal branch has four neighboring branches, which can represent a quartet topology. The dotted line indicates a one-third threshold expected at random. The top number of each subfigure indicates the label of the corresponding branch on the tree (right). Along the x-axis, the exact definition of each quartet topology is provided using neighboring branch labels separated by "|".

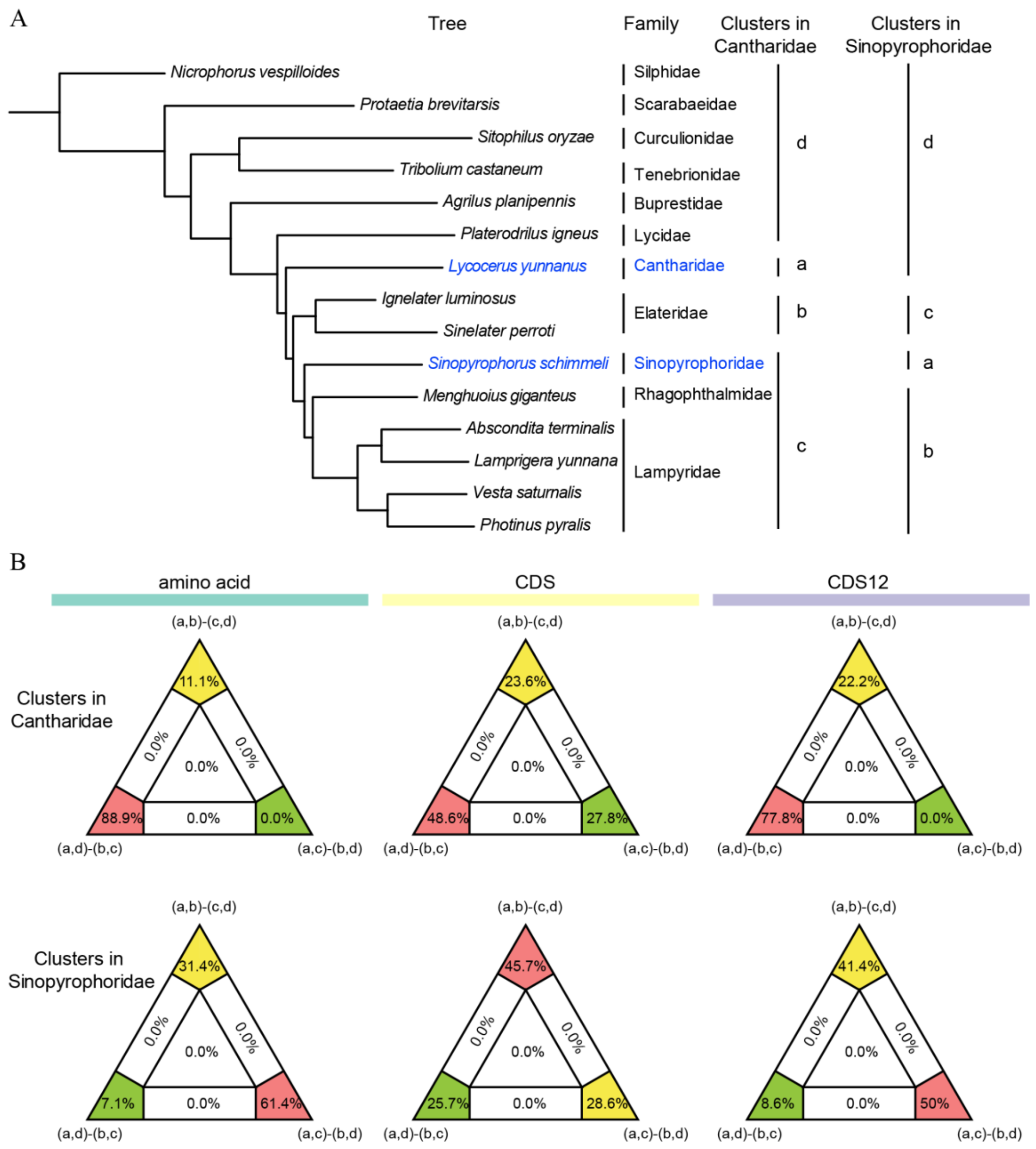

Supplementary fig. 7. Results of Four cluster likelihood mapping (FcLM) analyses for a selection of phylogenetic hypotheses applied at the amino acid and nucleotide levels using orthologous genes data matrix #1–#3. A. Species clustered into four groups. B. The test of the position of Cantharidae and Sinopyrophoridae.

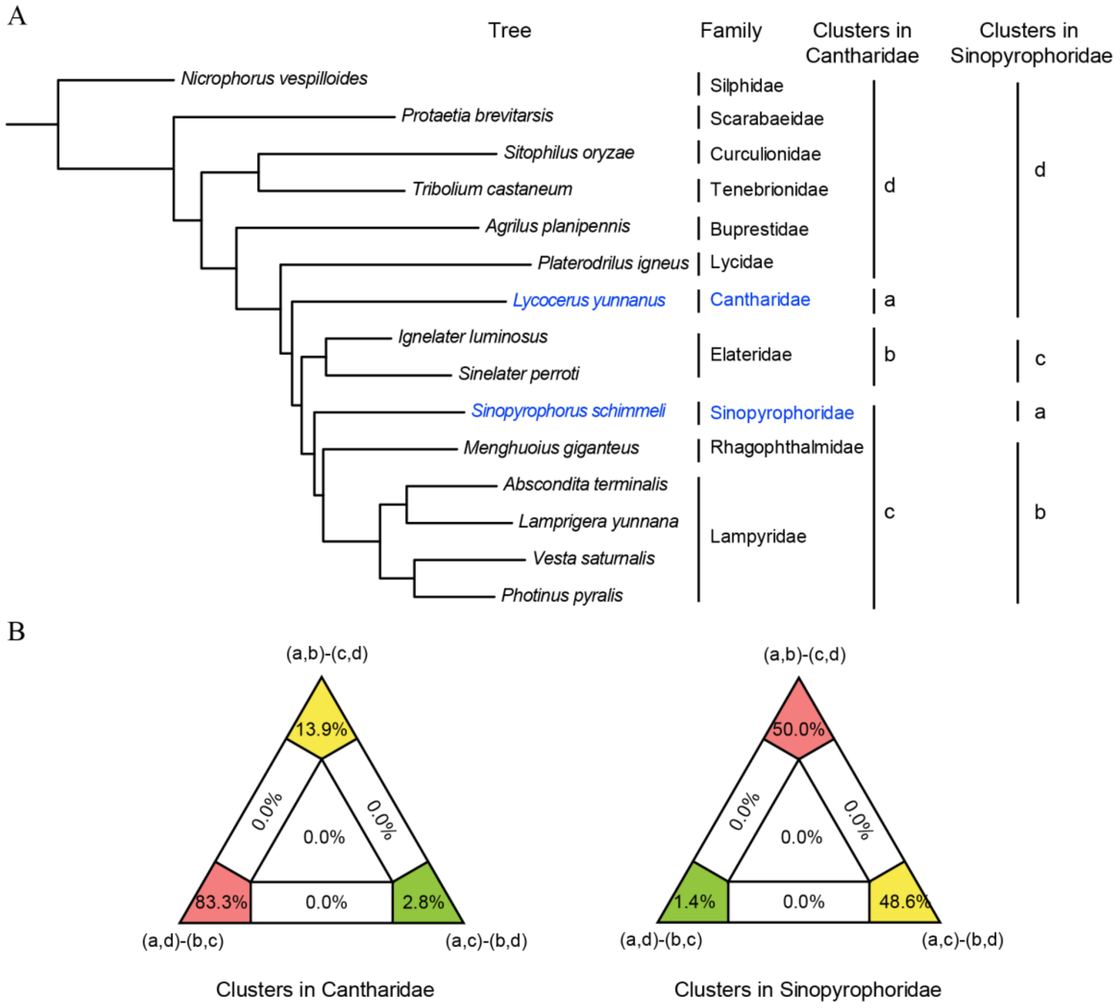

Supplementary fig. 8. Results of Four cluster likelihood mapping (FcLM) analyses for a selection of phylogenetic hypotheses applied at the amino acid level using BUSCO gene data matrix #4. A. Species clustered into four groups. B. The test of the position of Cantharidae and Sinopyrophoridae.

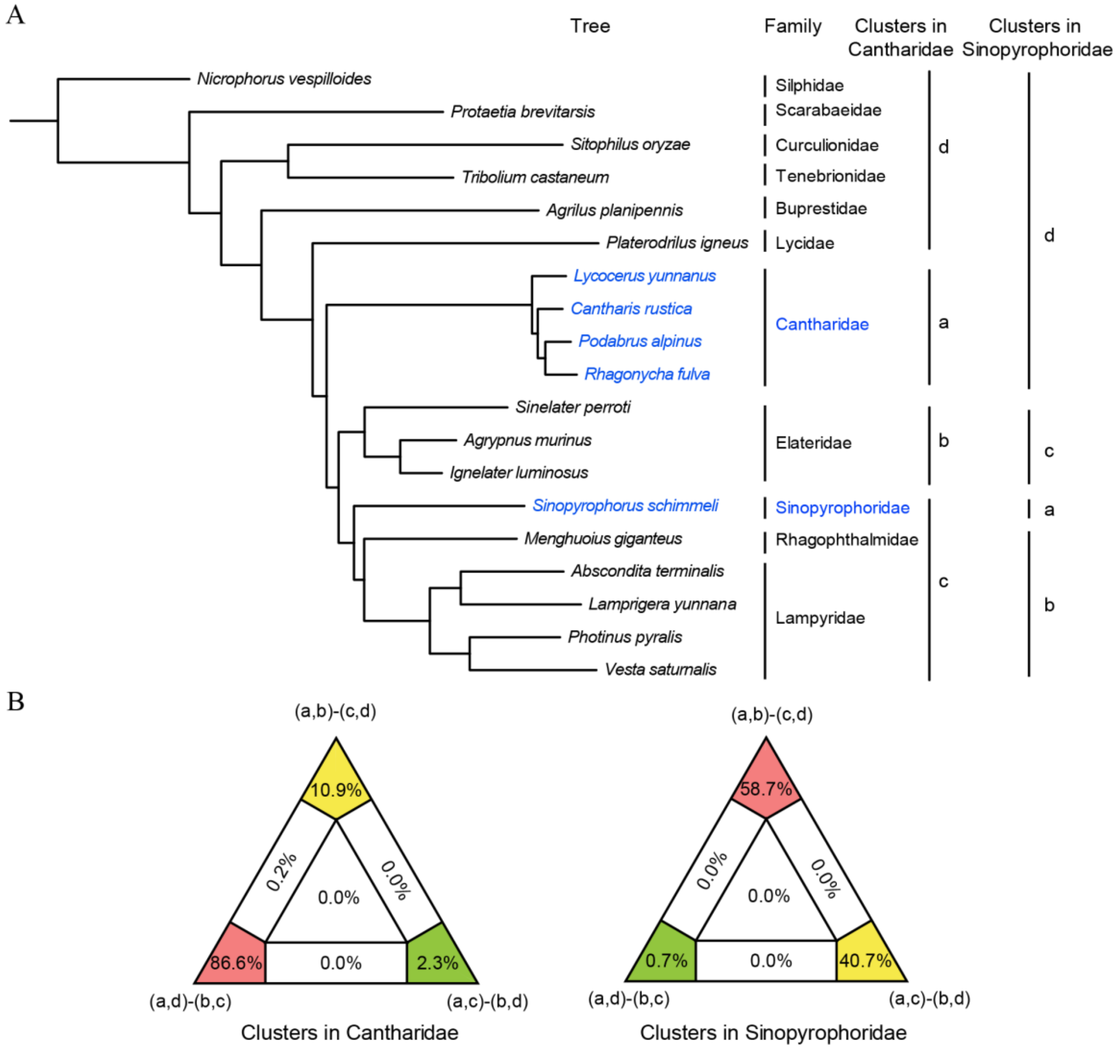

Supplementary fig. 9. Results of Four cluster likelihood mapping (FcLM) analyses for a selection of phylogenetic hypotheses applied at the amino acid level using BUSCO gene data matrix #5. A. Species clustered into four groups. B. The test of the position of Cantharidae and Sinopyrophoridae.

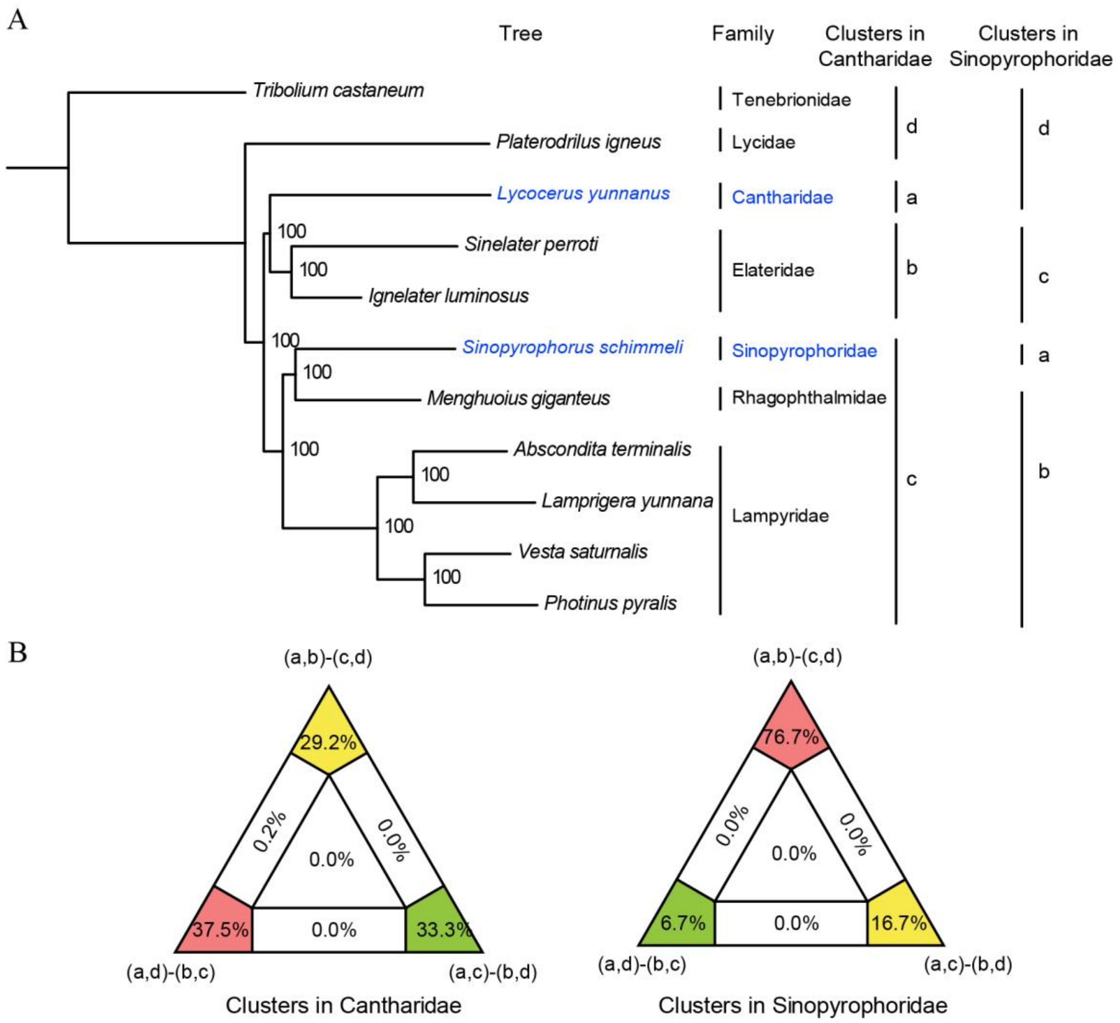

Supplementary fig. 10. Results of Four cluster likelihood mapping (FcLM) analyses for a selection of phylogenetic hypotheses applied at the nucleotide level using whole-genome alignment data matrix #6. A. Species clustered into four groups. B. The test of the position of Cantharidae and Sinopyrophoridae.

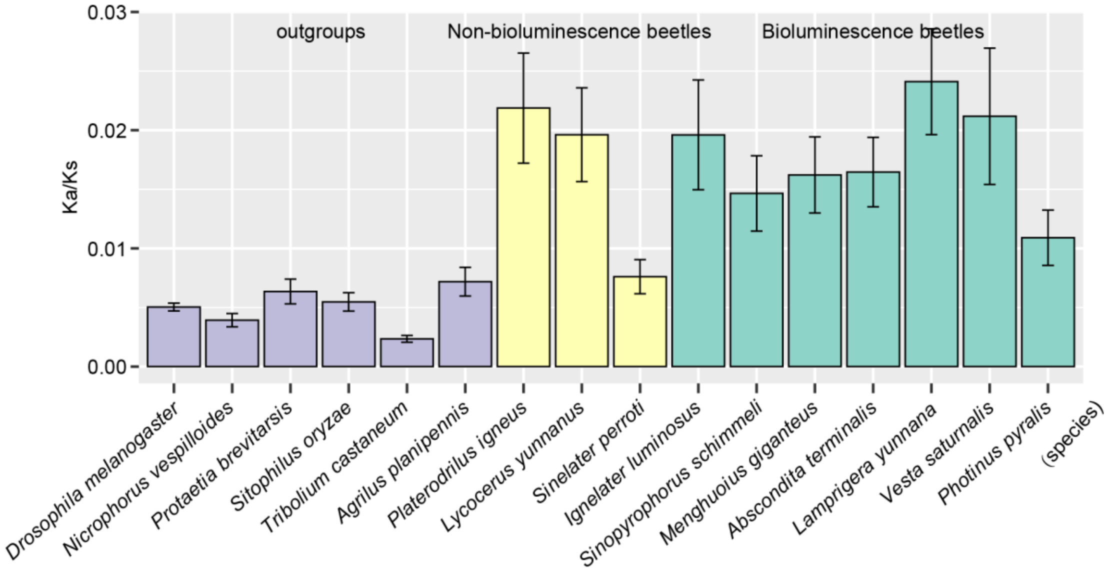

Supplementary fig. 11. Average *Ka*/*Ks* estimated using 10,000 concatenated alignments constructed based on 150 randomly chosen orthologs from the results of reciprocal BLAST analysis in OrthoFinder v2.4.0. Data represent mean ± standard error of the mean using 10,000 concatenated alignments.

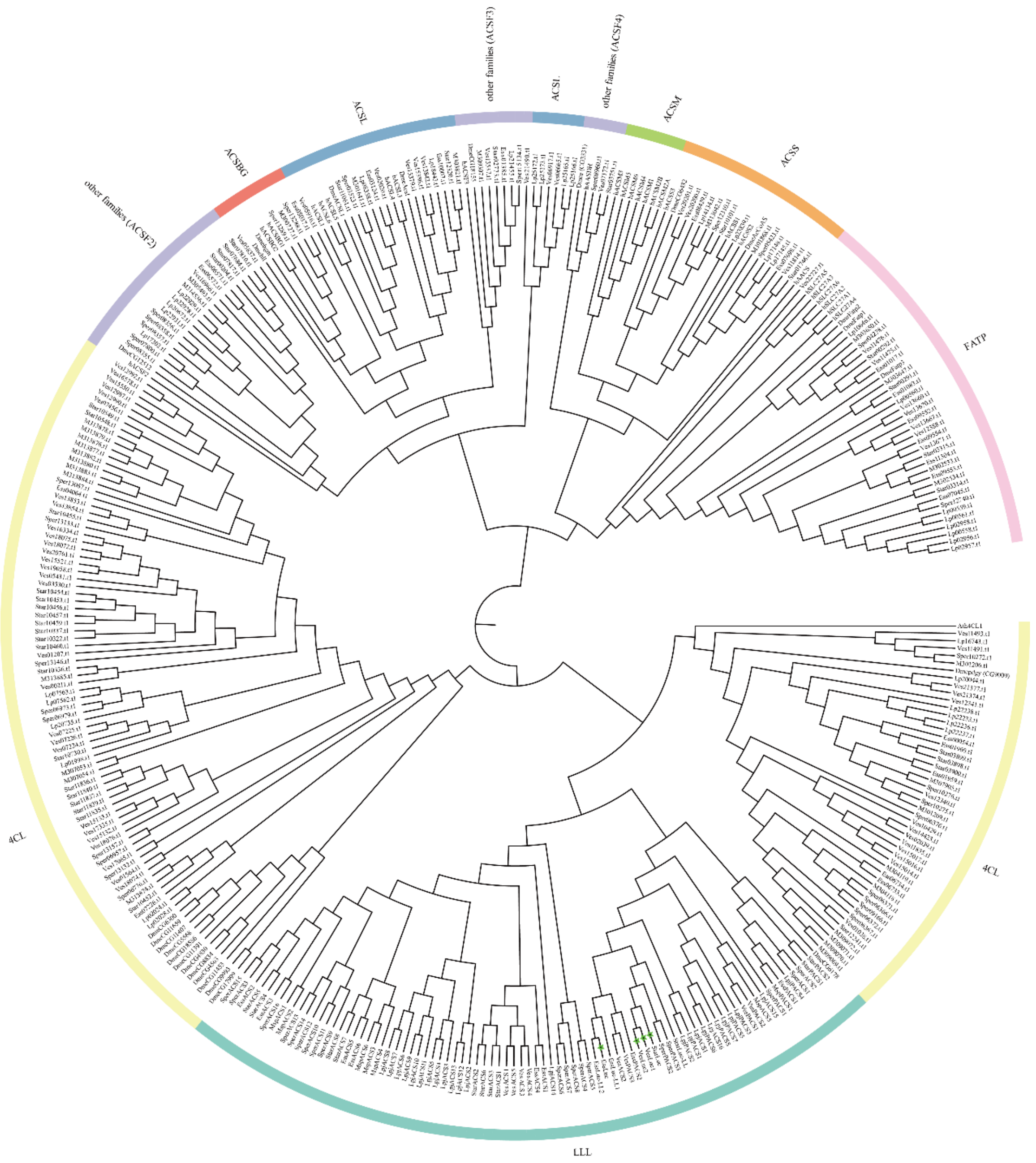

Supplementary fig. 12. Maximum likelihood (ML) tree and classification of acyl-CoA synthetase (ACS) superfamily in beetles, fruit fly, and human with the midpoint root. LLL (luciferases and luciferase-like genes), 4CL (4-coumarate: CoA ligases), ACSS (ACS short-chain family), ACSM (ACS medium-chain family), ACSBG (ACS bubblegum family), FATP (ACSVL, very long-chain ACS), ACSL (ACS long-chain family) and other families (ACSF2, ACS family member 2; ACSF3, ACS family member 3; AASDH (ACSF4)-aminoadipate-semialdehyde dehydrogenase). Green stars represent luciferases with bioluminescent activity.

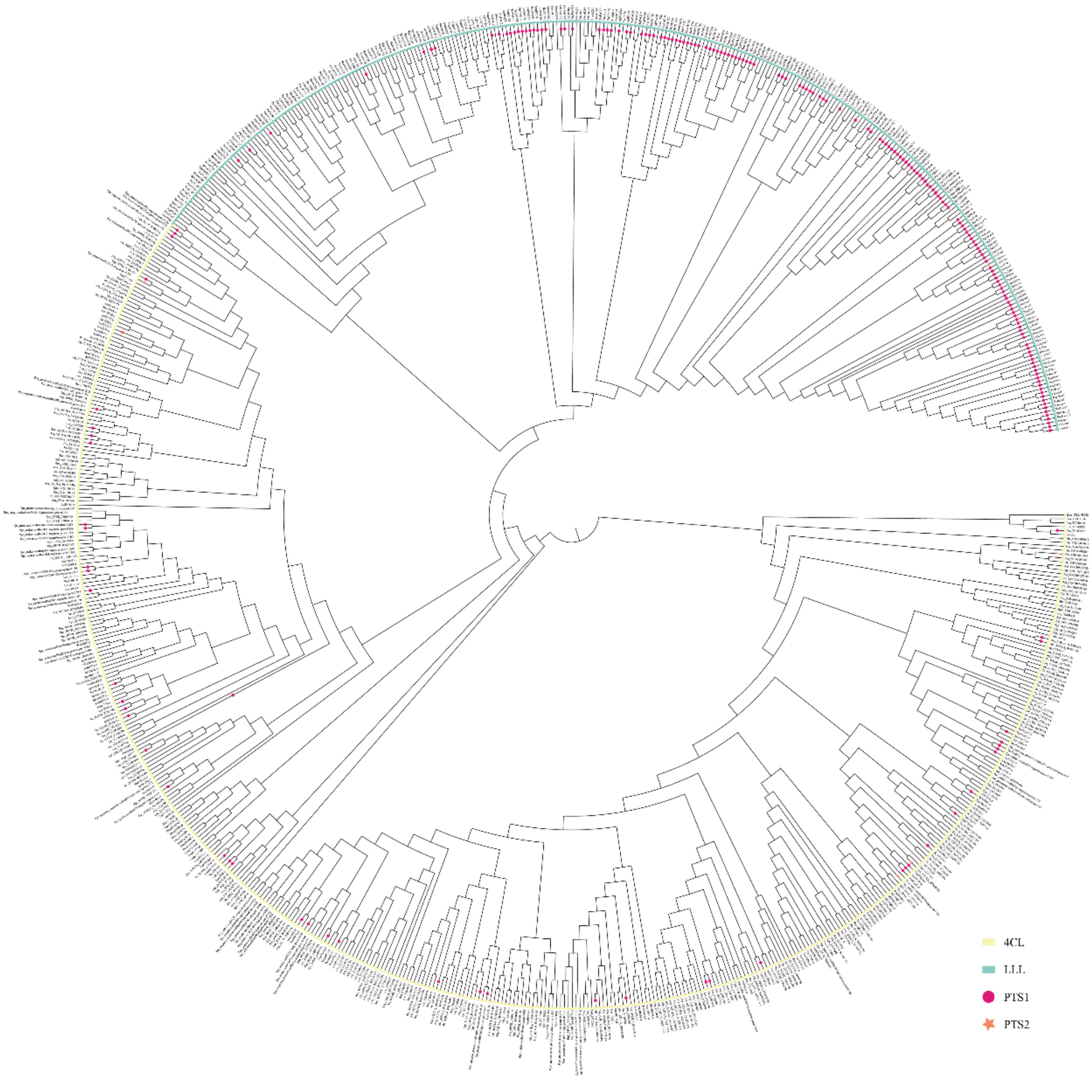

Supplementary fig. 13. Maximum likelihood (ML) tree of 4CL (4-coumarate: CoA ligases) and LLL genes (luciferase and luciferase-like genes) in beetles and fruit fly and cloned luciferase genes with the midpoint root. Red circles represent PTS1 (the C-terminal peroxisomal targeting signal 1), and stars represent PTS2 (the N-terminal peroxisomal targeting signal 2). The cloned luciferase genes showed in Supplementary data 10.

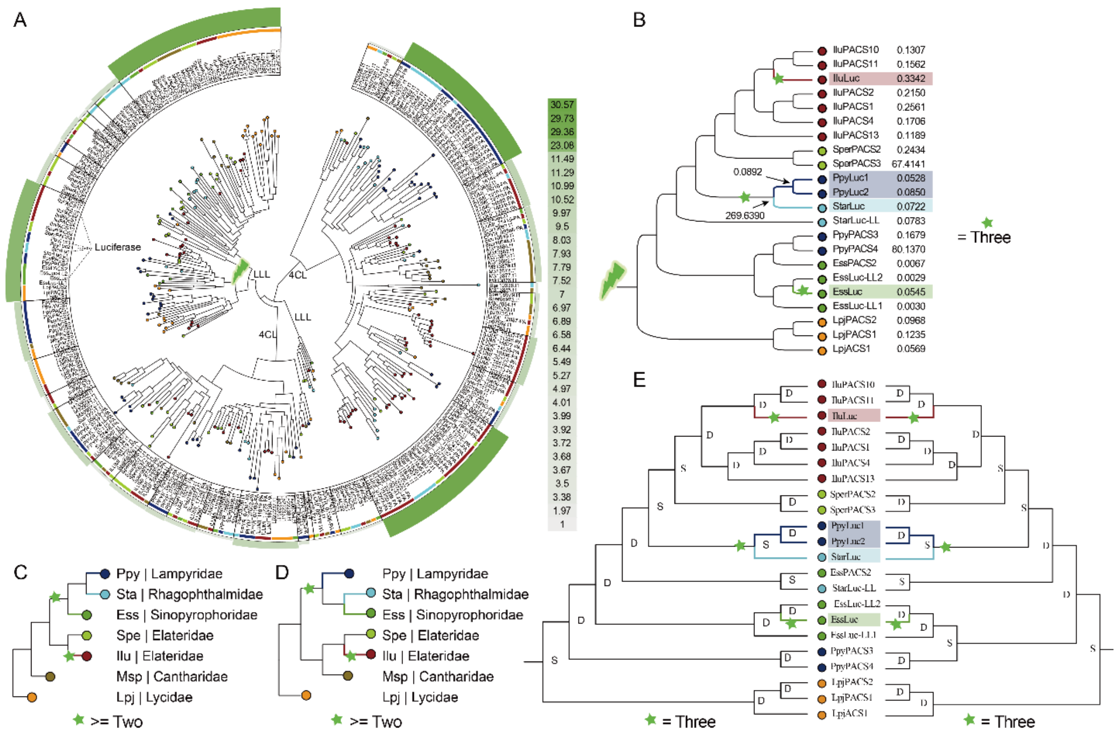

Supplementary fig. 14. Phylogenetic tree of 4-coumarate: CoA ligases (4CL) and luciferase and luciferase-like genes (LLL) in seven beetles. Green luminescent marker represents LLL subclade (including luciferases from all luminous families). Ppy: *Photinus pyralis*; Sta: *Menghuoius giganteus*; Ess: *Sinopyrophorus schimmeli*; Ilu: *Ignelater luminosus*; Msp: *Lycocerus yunnanus*; Lpj: *Platerodrilus igneus*. A. Phylogeny of 4CL and LLL genes using iqtree2 with the LG+F+R8 model. Instability score of each gene in the phylogenetic tree is represented by a color gradient, with darker green indicating a higher instability score. B. Substitution pattern of genes in LLL subclade containing luciferases. $\underline{\omega}$ values (*Ka*/*Ks*) based on number of synonymous (*Ks*) and nonsynonymous substitutions (*Ka*) are labeled on each branch. Luciferases highlighted with a colored background originated at least three times (green asterisk) in the gene tree. C. Input species tree derived from 568 single-copy orthologous genes for GeneRax. Bioluminescence originates at least two times (green asterisk). D. Input species tree derived from whole-genome alignments for GeneRax. Bioluminescence originated at least two times (green asterisk). E. Reconciled gene trees in LLL subclade using GeneRax. Corresponding species trees shown in C (left) and D (right), respectively. Luciferases highlighted with a colored background originated at least three times.

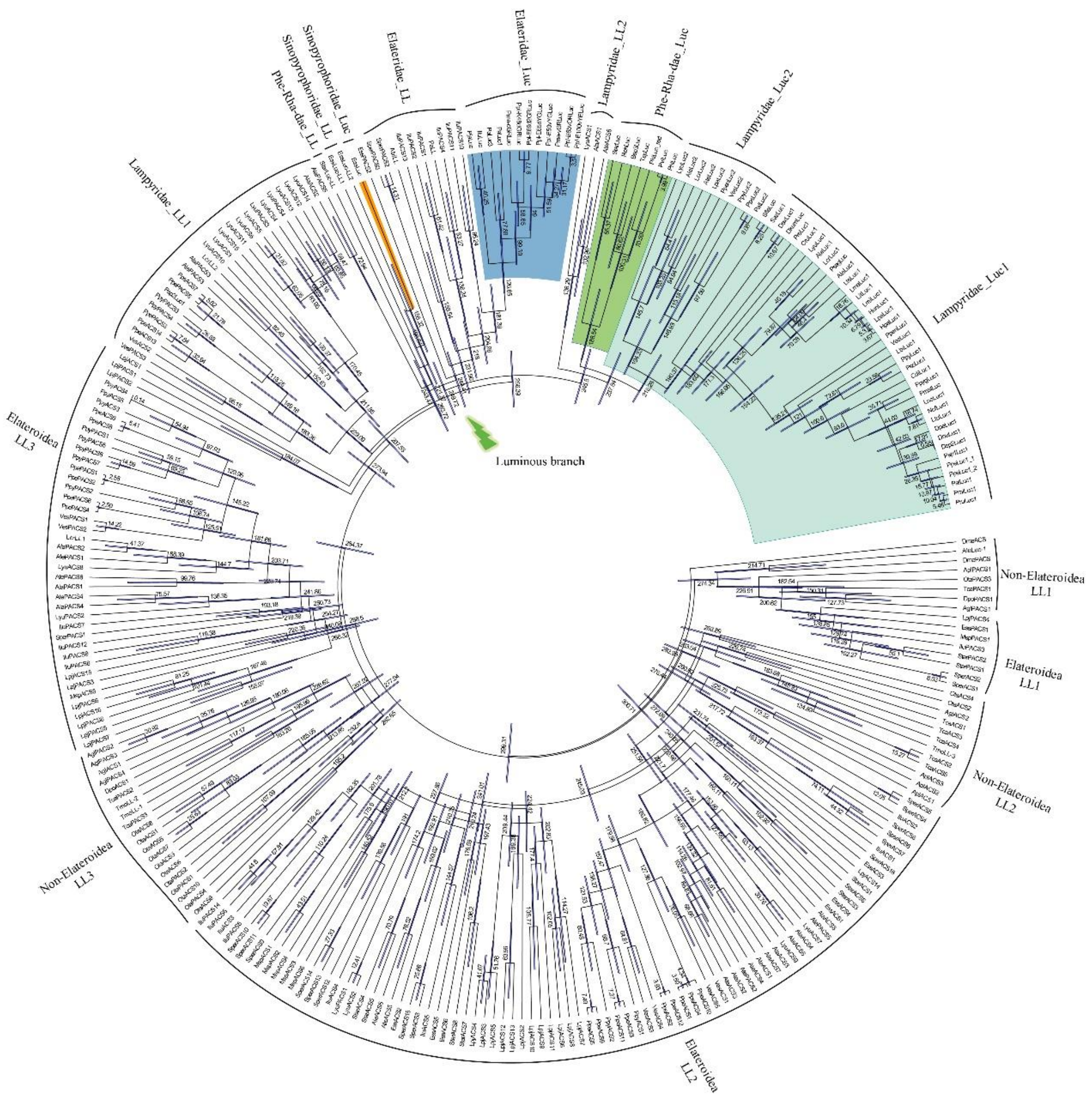

Supplementary fig. 15. Divergence times with 95% highest posterior density intervals of luciferase (Luc) and luciferase-like (LL) genes in beetles and fruit fly (unit: million years ago (Mya)). Divergence time was calculated using MCMCTREE and calibrated with the divergence time (~330 Mya) of the beetle and fruit fly (*D. melanogaster*: Dme) as a substitute for that of beetle LL and DmeACS.

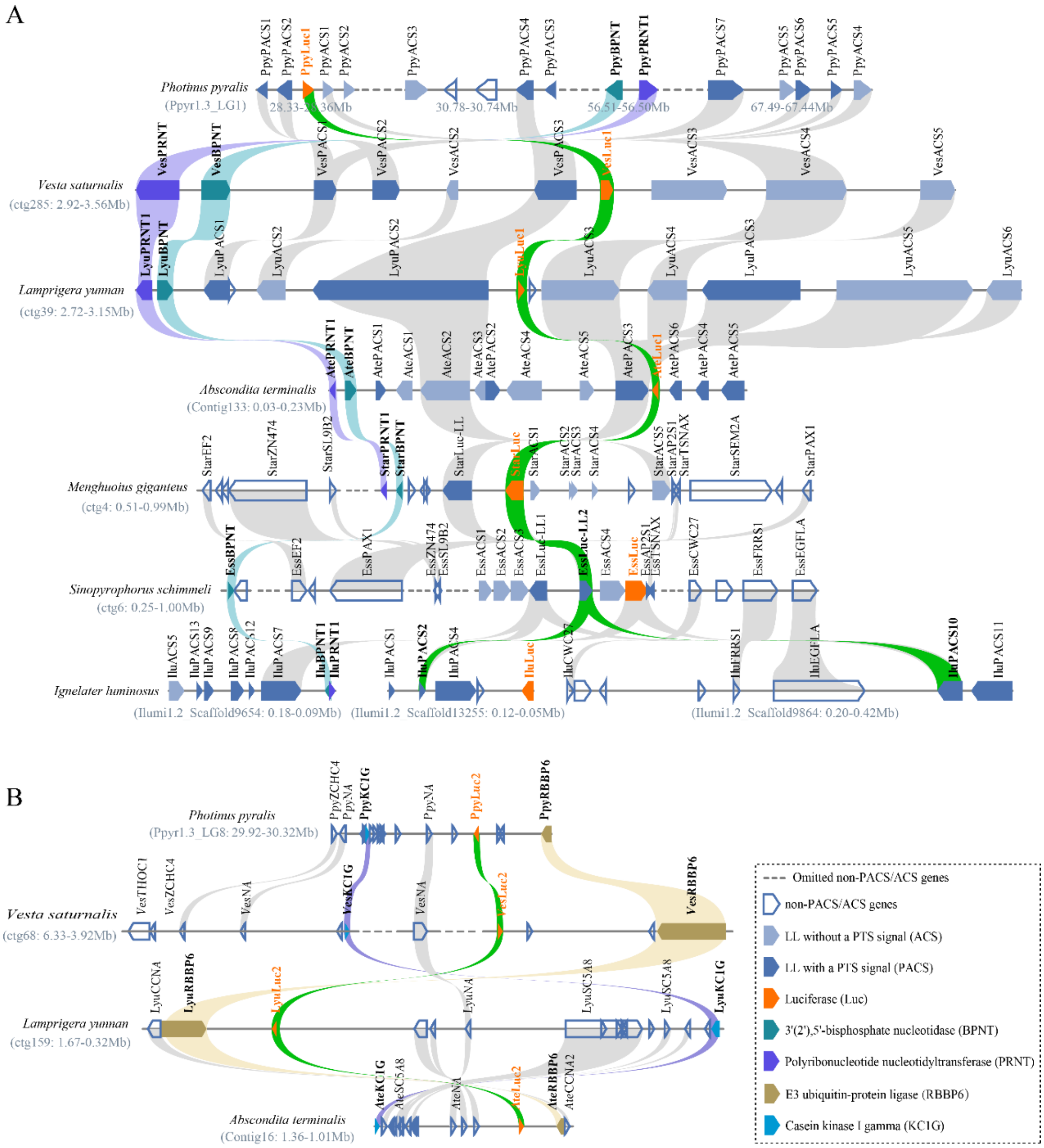

Supplementary fig. 16. Microsynteny analysis of syntenic block surrounding luciferases (Luc). Syntenic luciferase was highlighted in green. A. Microsynteny analysis of syntenic block surrounding luciferase (copy one in Lampyridae, *Luc1*) across seven luminous species. B. Microsynteny analysis of syntenic block surrounding luciferase (copy two in Lampyridae, *Luc2*) across four luminous species.

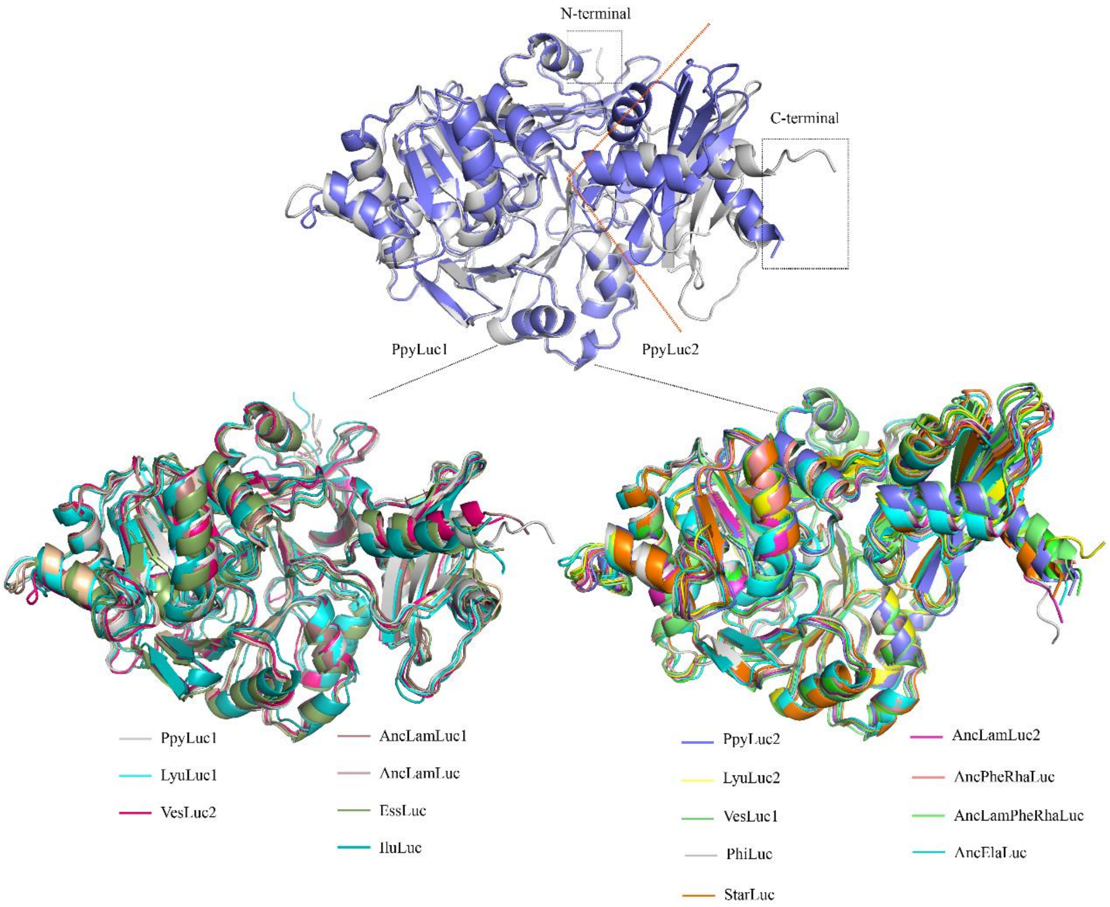

Supplementary fig. 17. Two distant three-dimensional structures of ancestral and extant luciferases predicted by AlphaFold2. Luciferases in the extant state including Ppy: *Photinus pyralis*; Lyu: *Lamprigera yunnana*; Ves: *Vesta saturnalis*; Star: *Menghuoius giganteus*; Phi: *Phrixothris hirtus*; Ess: *Sinopyrophorus schimmeli*; and Ilu: *Ignelater luminosus*. Luciferases in the ancestral state including AncLam: ancestral state of Lampyridae; AncPheRha: ancestral state of (Phengodidae + Rhagophthalmidae); AncLamPheRha: ancestral state of (Lampyridae + (Phengodidae + Rhagophthalmidae)); AncEla: ancestral state of Elateridae.

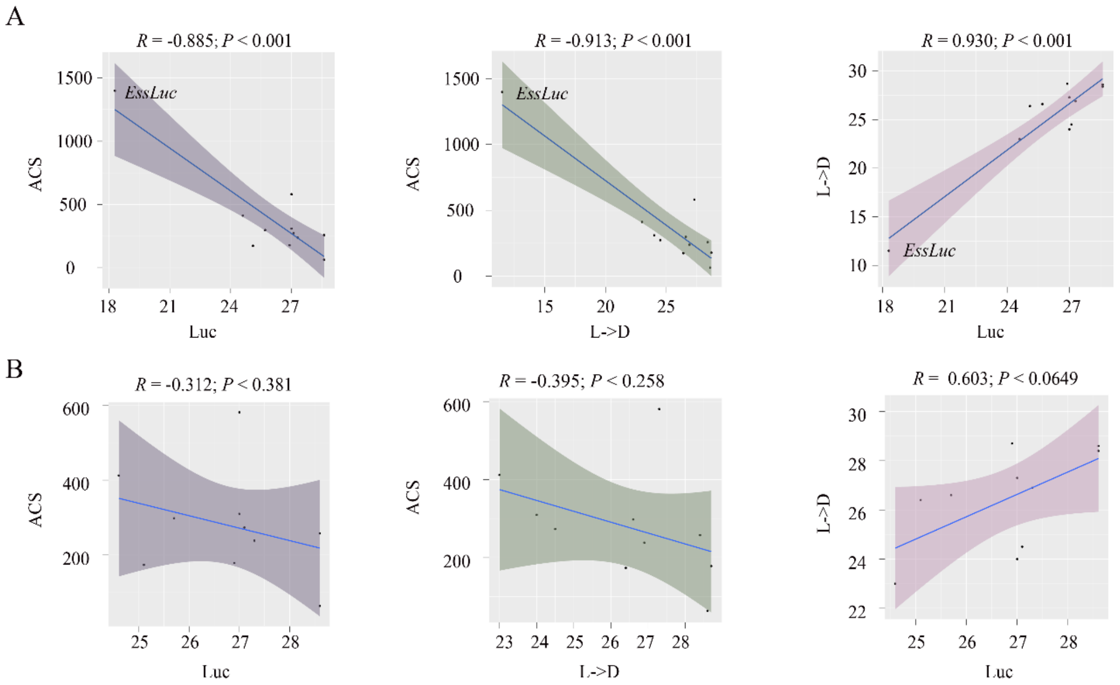

Supplementary fig. 18. Relationships between luminescent intensity (Luc), L- to D-luciferin transformation ability (L->D), and acyl-CoA synthetase activity (ACS) of reconstructed ancestral and extant luciferases using Pearson correlation analysis with R software. *R*: Pearson correlation coefficient; *P*: *P*-value. A and B are derived from datasets with and without *EssLuc*, respectively.

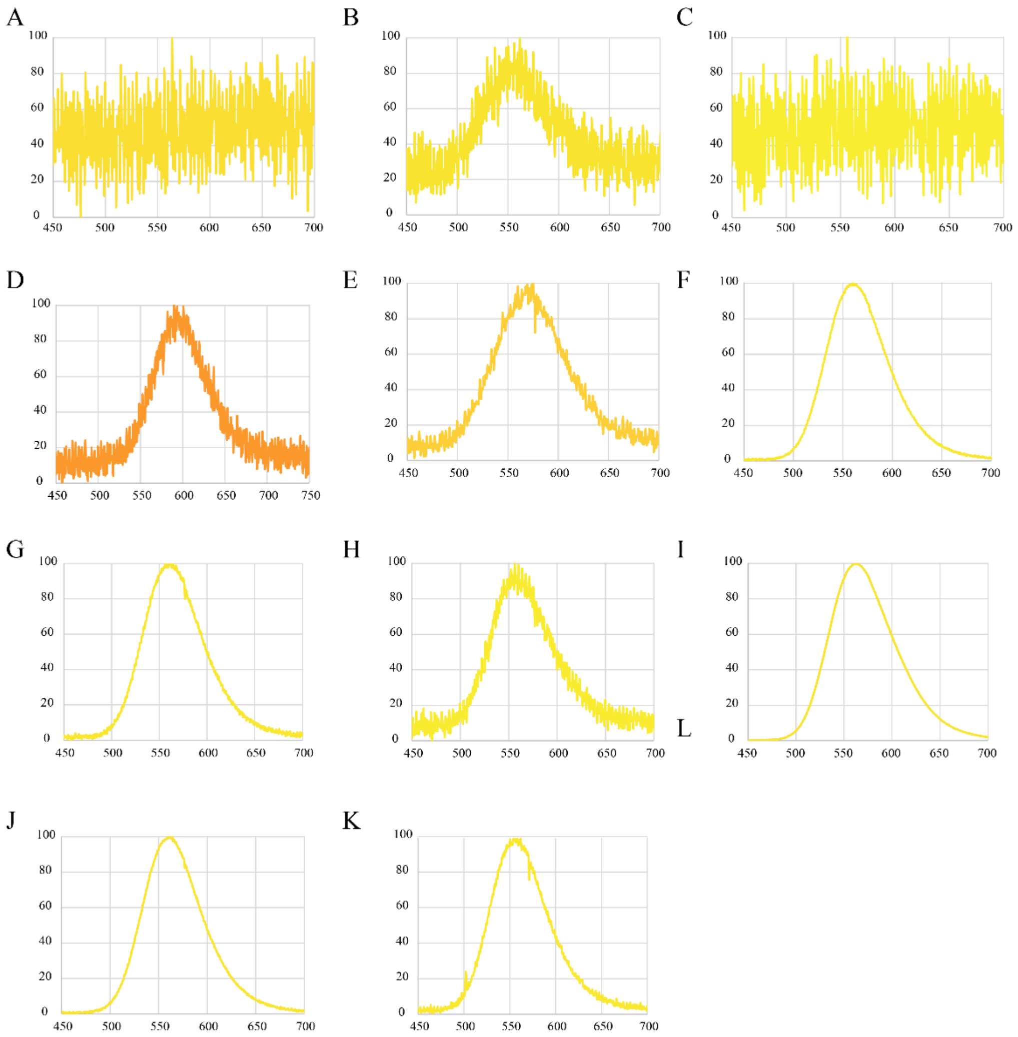

Supplementary fig. 19. Luminescent spectra of recombinant ancestral luciferase proteins and extant luciferase or luciferase-like proteins: A. *EssLuc*, B. AncElaLuc, C. *AteACS6*, D. AncLamPheRhaLuc, E. AncPheRhaLuc, F. AncLamLuc, G. AncLamLuc2, H. *VesLuc2*, I. AncLamLuc1, J. *VesLuc1*, and K. *LyuLuc1*, respectively.

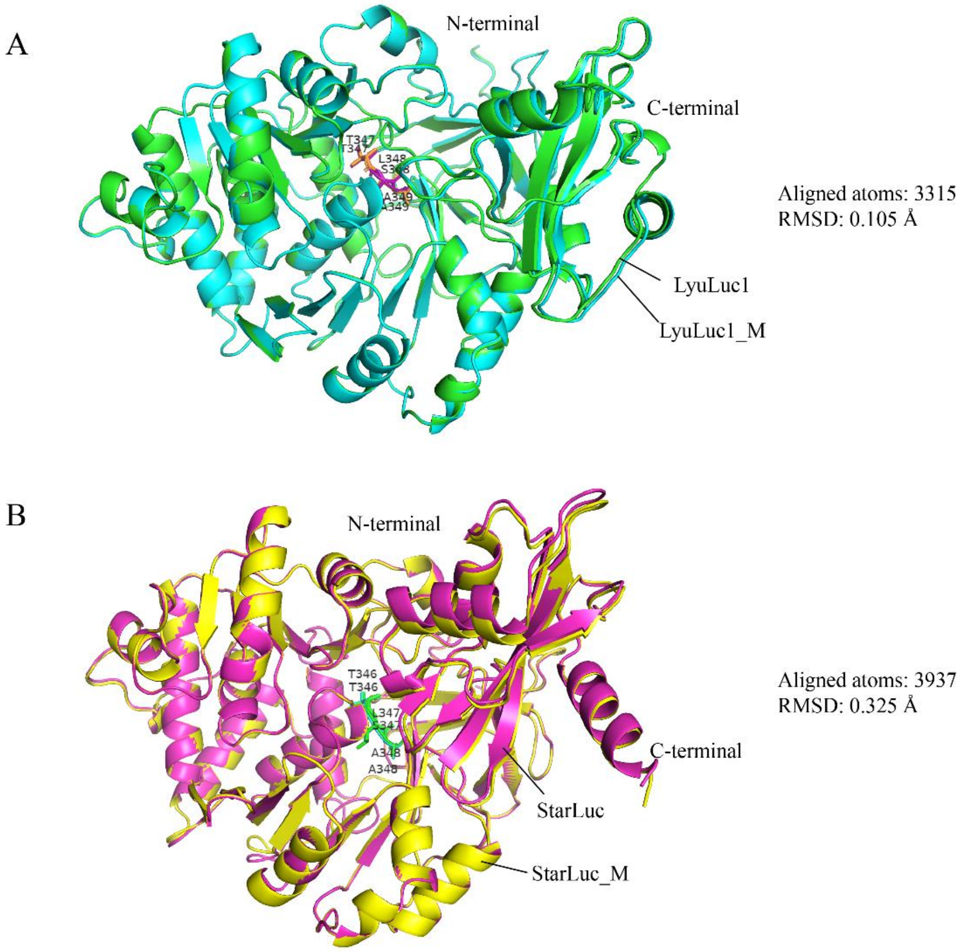

Supplementary fig. 20. Three-dimensional structures of the wild-types and mutants of luciferases from A. *Lamprigera yunnana* (Lyu: Lampyridae) and B. *Menghuoius giganteus* (Star: Rhagophthalmidae). Luciferin-binding sites (LBSs) showed as a stick with labels.

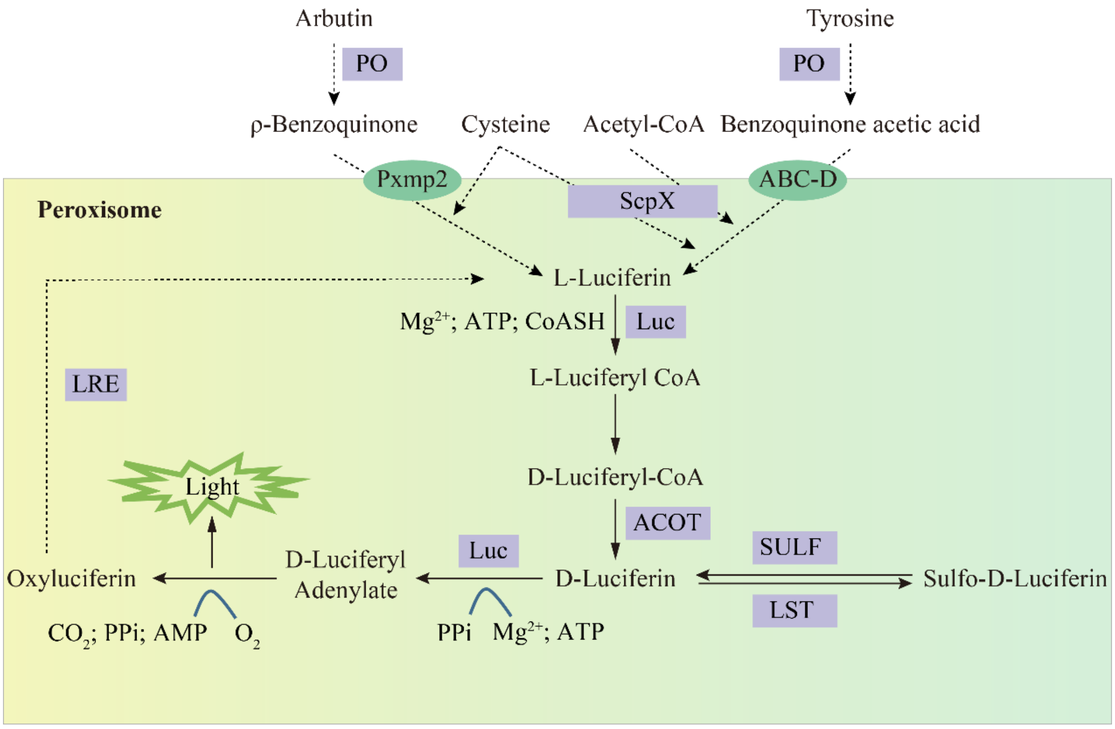

Supplementary fig. 21. Pathway of luciferin biosynthesis simplified from Zhang et al (2020). PO: phenoloxidase; Pxmp2: peroxisomal membrane protein 2; ABC-D: ATP-binding cassette protein D; ScpX: sterol carrier protein; Luc: luciferase; ACOT: acyl-CoA thioesterases; SULF: sulfatase; LST: luciferin sulfotransferase; LRE: luciferin-regenerating enzyme.

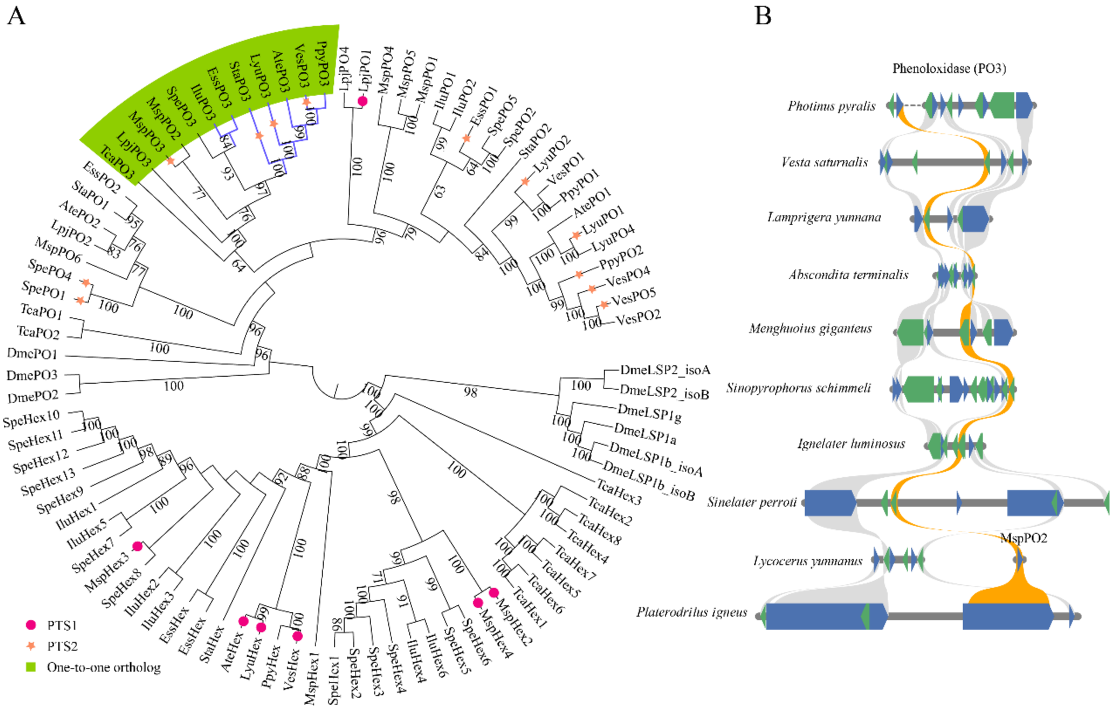

Supplementary fig. 22. Evolution of phenoloxidases (POs). A. Gene tree of PO and hexamerin (Hex) was inferred using IQ-TREE. Red circles represent C-terminal peroxisomal targeting signal 1 (PTS1), and stars represent N-terminal peroxisomal targeting signal 2 (PTS2). B. Syntenic relationships of one-to-one PO orthologs among ten beetles. Syntenic PO was highlighted in yellow.

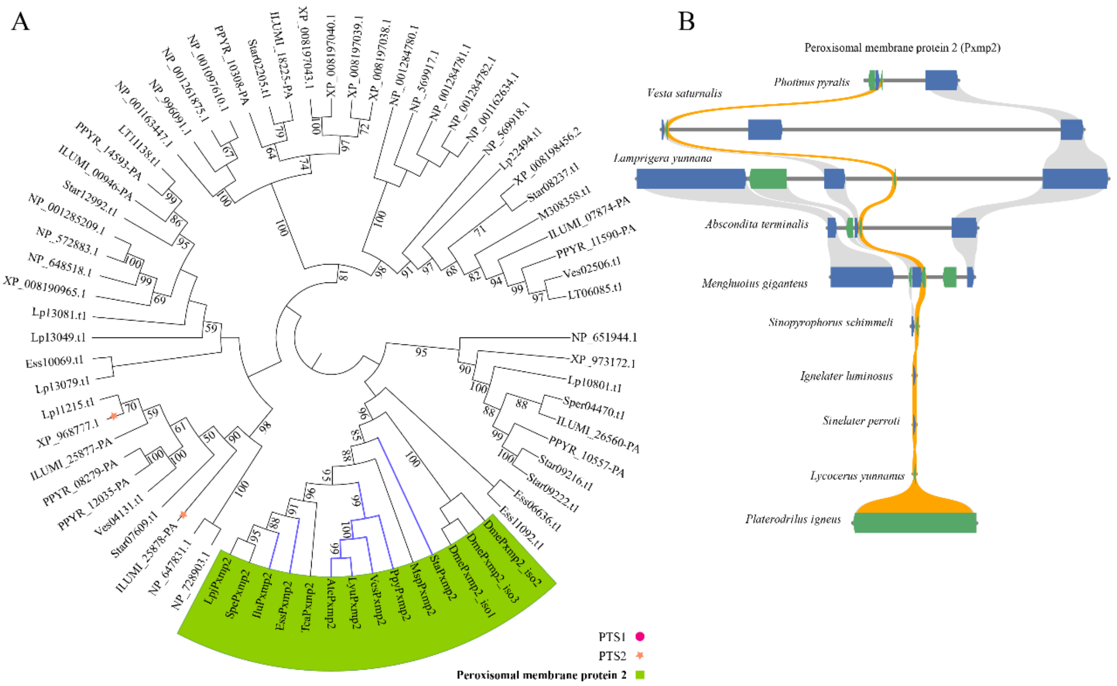

Supplementary fig. 23. Evolution of peroxisomal membrane protein 2s (Pxmp2s).

A. Gene tree of Pxmp2s was inferred using IQ-TREE. Red circles represent C-terminal peroxisomal targeting signal 1 (PTS1), and stars represent N-terminal peroxisomal targeting signal 2 (PTS2). B. Syntenic relationships of one-to-one Pxmp2 orthologs among ten species. Syntenic Pxmp2 was highlighted in yellow.

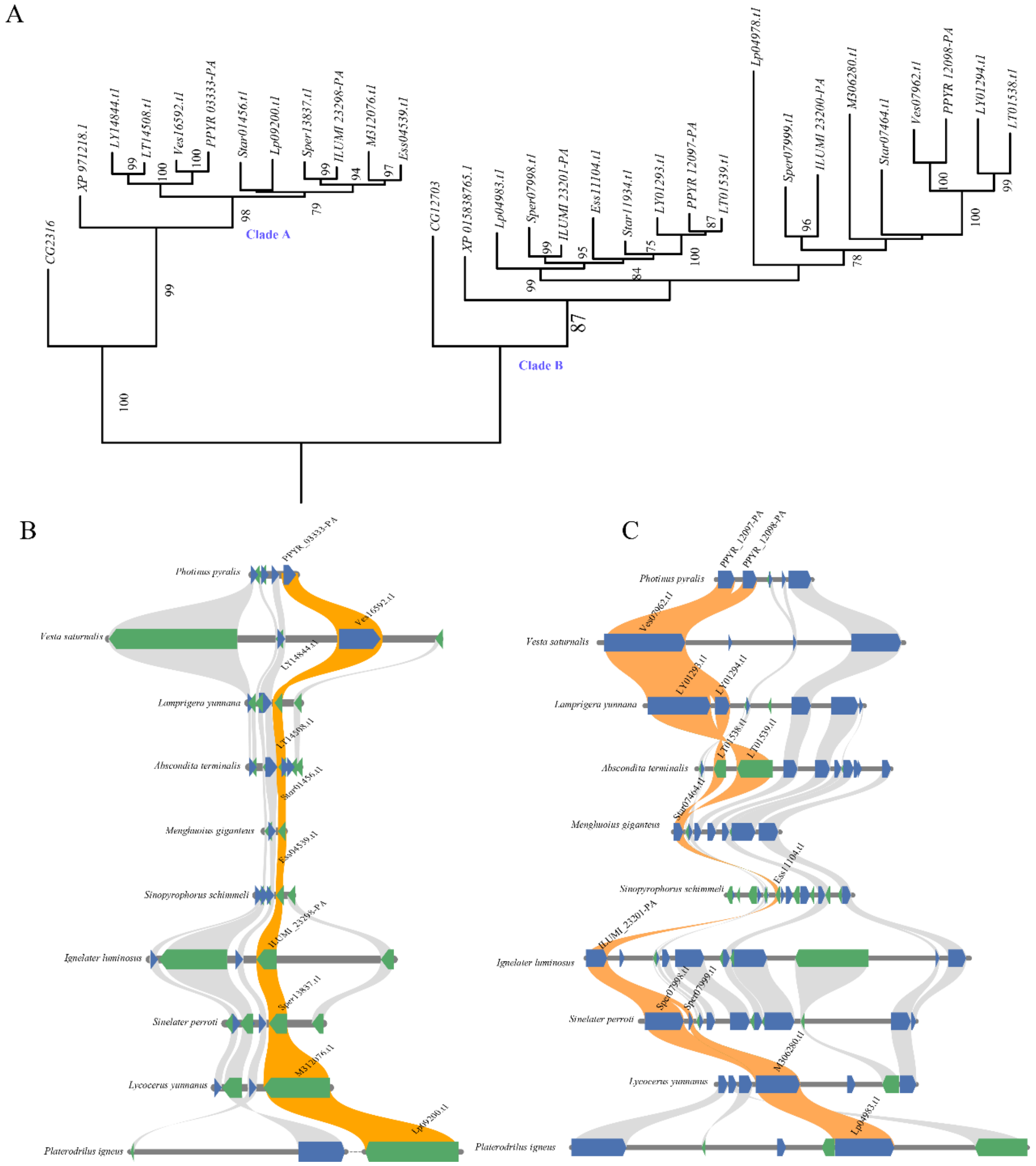

Supplementary fig. 24. Evolution of ATP-binding cassette protein Ds (ABC-Ds). Syntenic ABC-D was highlighted in yellow. A. Gene tree of ABC-Ds was inferred using IQ-TREE. B. Syntenic relationships of ABC-D in clade A among ten beetles. C. Syntenic relationships of ABC-D in clade B among ten beetles.

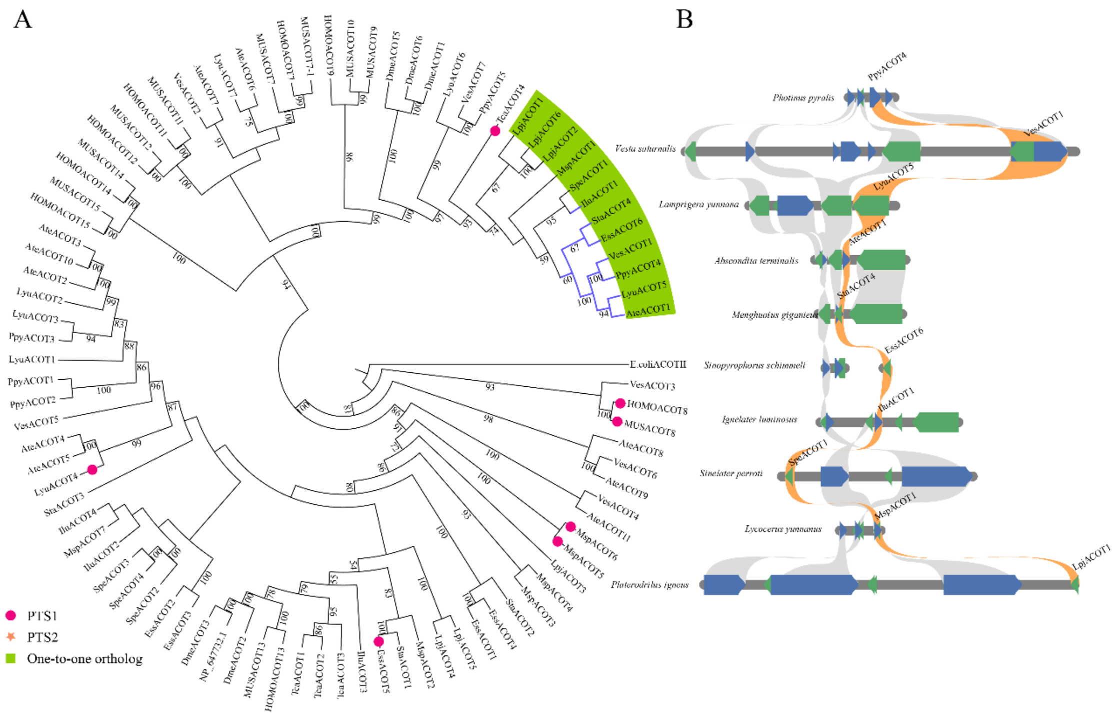

Supplementary fig. 25. Evolution of acyl-CoA thioesterases (ACOTs). Red circles represent C-terminal peroxisomal targeting signal 1 (PTS1), and stars represent N-terminal peroxisomal targeting signal 2 (PTS2). A. Gene tree of ACOTs was inferred using IQ-TREE. B. Syntenic relationships of one-to-one ACOT orthologs among ten beetles. Syntenic ACOT was highlighted in yellow.

A

B

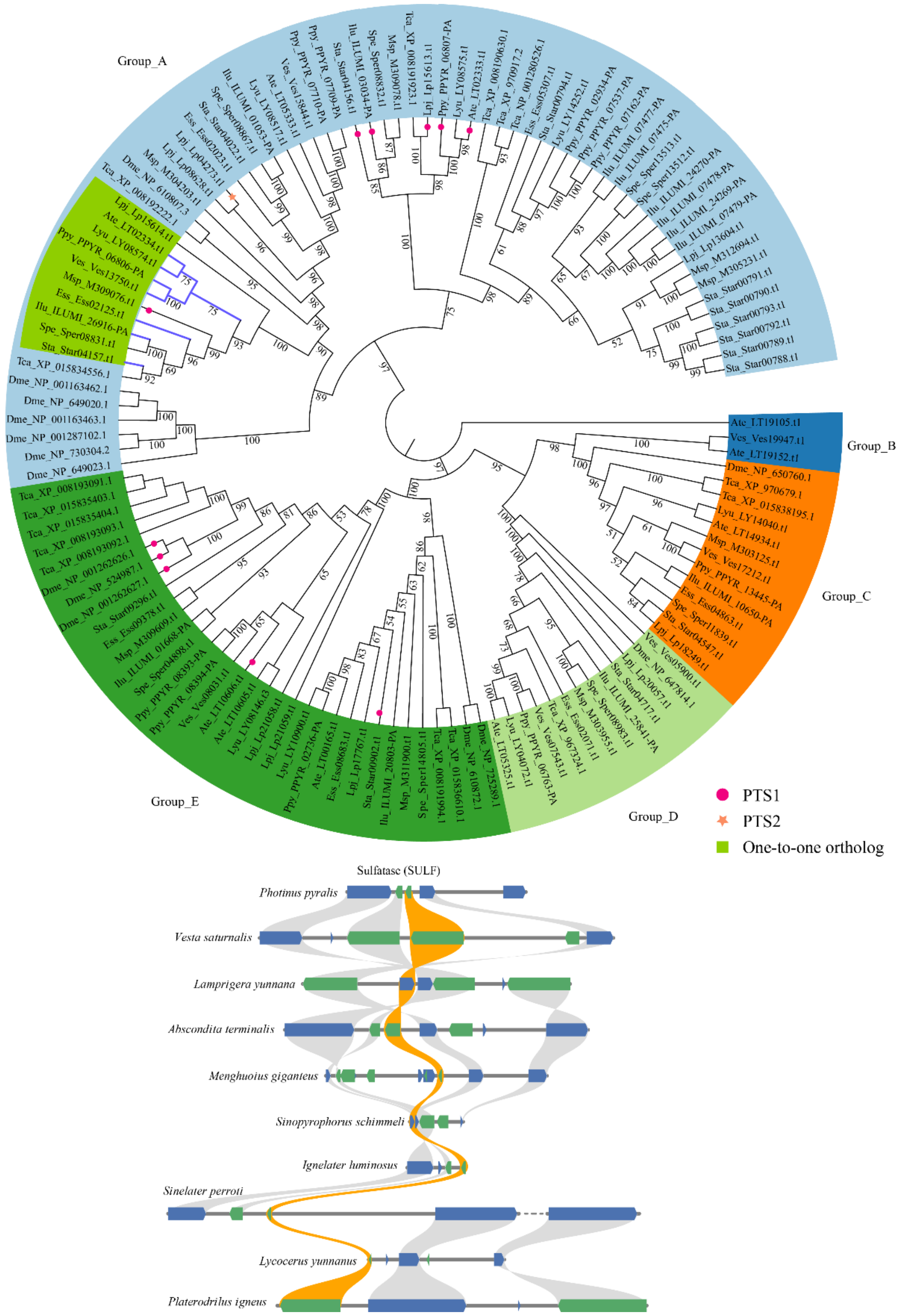

Supplementary fig. 26. Evolution of sulfatases (SULFs). Red circles represent C-terminal peroxisomal targeting signal 1 (PTS1), and stars represent N-terminal peroxisomal targeting signal 2 (PTS2). A. Gene tree of SULFs was inferred using IQ-TREE. B. Syntenic relationships of one-to-one SULF orthologs among ten beetles. Syntenic SULF was highlighted in yellow.

A B

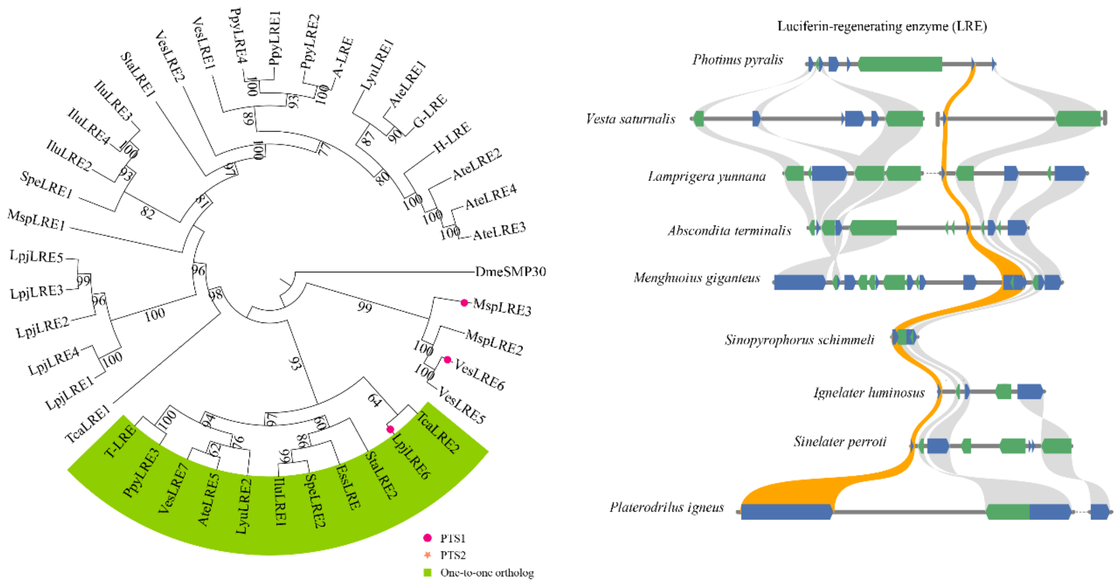

Supplementary fig. 27. Evolution of luciferin-regenerating enzymes (LREs). Red circles represent C-terminal peroxisomal targeting signal 1 (PTS1), and stars represent N-terminal peroxisomal targeting signal 2 (PTS2). A. Gene tree of LRE was inferred using IQ-TREE. B. Syntenic relationships of one-to-one LRE orthologs among nine beetles. Syntenic LRE was highlighted in yellow.

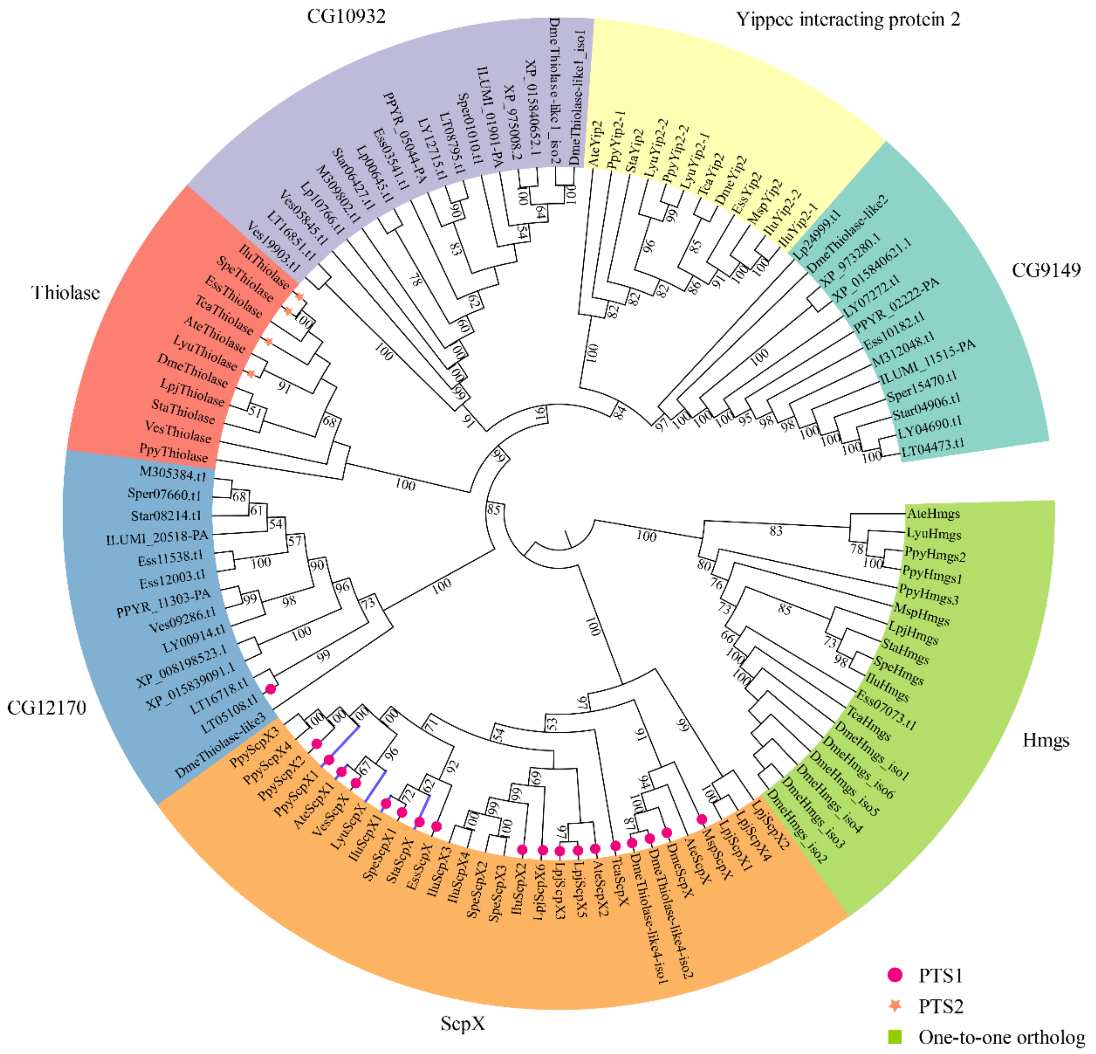

Supplementary fig. 28. Gene tree of Thiolases (ScpXs) was inferred using IQ-TREE. Red circles represent C-terminal peroxisomal targeting signal 1 (PTS1), and stars represent N-terminal peroxisomal targeting signal 2 (PTS2).

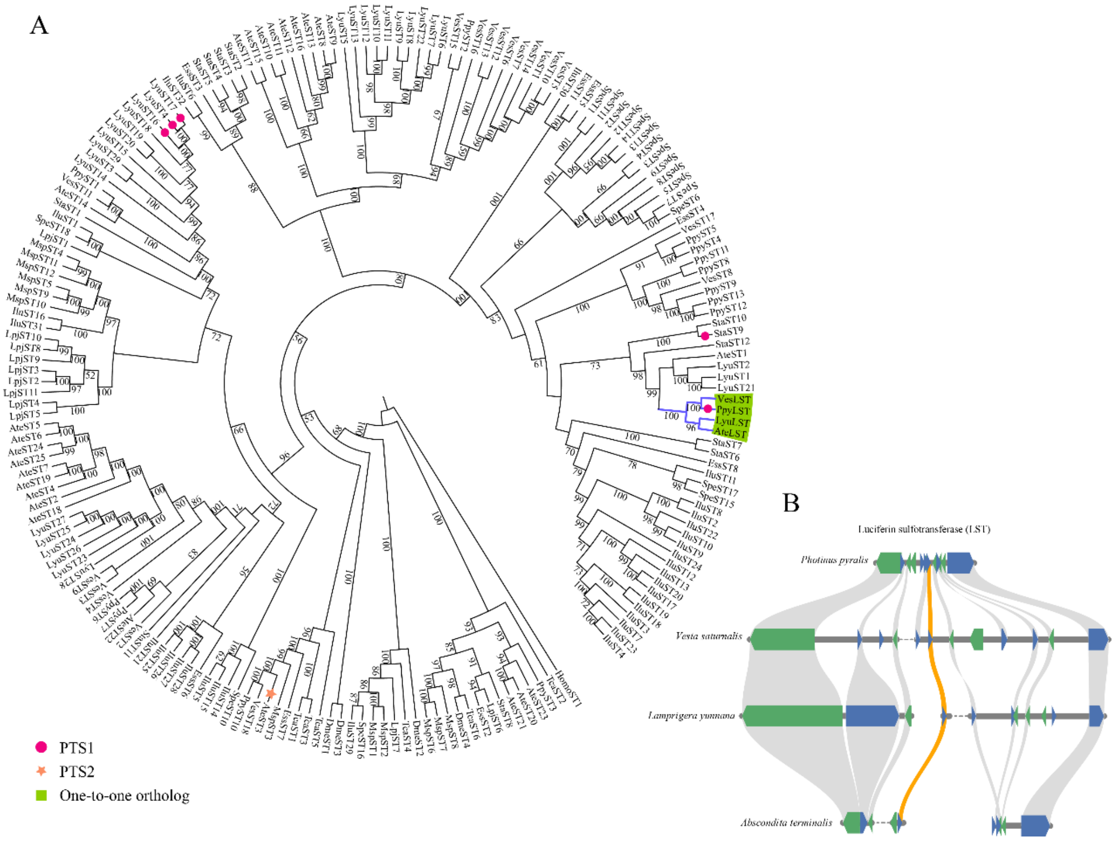

Supplementary fig. 29. Evolution of luciferin sulfotransferases (LSTs). Red circles represent C-terminal peroxisomal targeting signal 1 (PTS1), and stars represent N-terminal peroxisomal targeting signal 2 (PTS2). A. Gene tree of LST was inferred using IQ-TREE. B. Syntenic relationships of one-to-one LST orthologs among four fireflies. Syntenic LST was highlighted in yellow.

Supplementary tables:

Supplementary table 1. Summary of gene function annotation for the assembled genomes in this study.

Database	***Platerodrilus igneus***		***Lycocerus yunnanus***		***Sinelater perroti***		***Sinopyrophorus schimmeli***		***Menghuoius giganteus***		***Vesta saturnalis***	
	Number	Percent (%)	Number	Percent (%)	Number	Percent (%)	Number	Percent (%)	Number	Percent (%)	Number	Percent (%)
**Prediction**	**25,393**	**-**	**18,811**	**-**	**16,302**	**-**	**12,233**	**-**	**13,046**	**-**	**22,921**	**-**
InterPro	16,747	65.95	8,228	43.76	10,418	63.95	8,497	69.47	10,077	77.25	14,491	63.18
GO	9,695	38.18	5,648	30.04	7,140	43.83	6,009	49.13	7,088	54.34	8,712	37.99
KEGG	11,529	45.40	11,101	59.04	8,786	53.93	7,777	63.58	7,954	60.98	9,642	42.04
Swissprot	13,302	52.39	12,552	66.76	10,096	61.97	9,103	74.43	9,282	71.16	10,954	47.76
TrEMBL	21,808	85.89	15,860	84.35	12,964	79.58	10,772	88.07	11,492	88.10	19,556	85.27
NR	21,789	85.81	16,098	85.61	13,083	80.31	10,859	88.78	11,598	88.91	19,692	85.86
**Annotated**	**22,513**	**88.66**	**16,428**	**87.33**	**13,379**	**82.07**	**10,999**	**89.93**	**11,750**	**90.07**	**20,188**	**88.01**
Unannotated	2,879	17.19	2,383	28.96	2,923	28.06	1,232	14.50	1,295	12.85	2,751	18.98

Supplementary table 2. Residues near oxyluciferin 3.5 Å in complexes.

PpyLuc1	**218**	**245**	**246**	**247**	**314**	**315**	**316**	**317**	**318**	**319**	**337**	**338**	**339**	**340**	**341**	**342**	**343**	**346**	**347**	**348**	**360**	**362**	**433**	**434**	**437**	**443**	**448**
**position**	**R**	**H**	**G**	**F**	**S**	**G**	**G**	**A**	**P**	**L**	**R**	**Q**	**G**	**Y**	**G**	**L**	**T**	**T**	**S**	**A**	**G**	**V**	**F**	**I**	**R**	**K**	**Q**
LyuLuc1	.	.	.	.	.	.	.	.	.	.	.	.	.	.	.	.	.	.	.	.	.	C	.	.	.	.	.
LyuLuc1_M	.	.	.	.	.	.	.	.	.	.	.	.	.	.	.	.	.	.	L	.	.	C	.	.	.	.	.
StarLuc	.	.	.	.	T	.	.	.	.	V	L	.	.	.	.	.	.	C	C	.	.	T	Y	V	.	.	.
StarLuc_M	.	.	.	.	T	.	.	.	.	V	L	.	.	.	.	.	.	.	L	.	.	T	Y	V	.	.	.

Green: 3.5 Å residues; Yellow: Hydrogen bonded residues and oxyluciferin; Red: Mutated sites.

Supplementary table 3. Reconstructed ancestral luciferases.

**Ancestral luciferase**	**Ancestor position**	**Av. P.P.***
AncLamLuc1	The common ancestor of copy one luciferases (*Luc1*) of Lampyridae	0.97005
AncLamLuc2	The common ancestor of copy two luciferases (*Luc2*) of Lampyridae	0.93931
AncLamLuc	The common ancestor of luciferases of Lampyridae	0.92512
AncPheRhaLuc	The common ancestor of luciferases of the Phengodidae-Rhagophthalmidae lineage	0.91363
AncLamPheRhaLuc	The common ancestor of luciferases of the Lampyridae-Phengodidae-Rhagophthalmidae lineage	0.91195
AncElaLuc	The common ancestor of luciferases of Elateridae	0.97073

*Av. P.P.: average posterior probability of the amino acid residues.

Supplementary table 4. Primer sequences for the amplification of the luciferase (Luc) and luciferase-like (LL) genes of *S. schimmeli*.

Gene name	Primer Name	Sequence (5’ to 3’)
Ess02250	Ess02250_F1	CTACATATGATGTCCGATTCGACTAA
(*EssLuc-LL2*)	Ess02250_R1	CCGCTCGAGGTACGACTACAAAAGCG
Ess02252	Ess02252_F1	CTACATATGATGGATAGTAATATACT
(*EssLuc*)	Ess02252_R1	CCGCTCGAGTTAACACCACAAAAGCT

Legends for supplementary data

Supplementary data 1. Full information of species used in this study.

Supplementary data 2. Information on genomic sequencing of six species in this study.

Supplementary data 3. Enrichment of gene families in each common ancestral branch of luminous family (AB-ELF, AB-LRSE, AB-LRS, AB-LR, AB-Lam, AB-Ela), luminous species (Ilu, Ess, Sta), and non-luminous species (Lpj, Msp, Spe).

Supplementary data 4. Rapid evolution genes (REGs) in each common ancestral branch of luminous family (AB-ELF, AB-LRSE, AB-LRS, AB-LR, AB-Lam, AB-Ela), luminous species (Ilu, Ess, Sta), and non-luminous species (Lpj, Msp, Spe).

Supplementary data 5. Positively selected genes (PSGs) in each common ancestral branch of luminous family (AB-ELF, AB-LRSE, AB-LRS, AB-LR, AB-Lam, AB-Ela), luminous species (Ilu, Ess, Sta), and non-luminous species (Lpj, Msp, Spe).

Supplementary data 6. Enrichment of rapid evolution genes (REGs) each common ancestral branch of luminous family (AB-LRSE, AB-LRS, AB-LR, AB-Lam, AB-Ela) and luminous species (Sta, Ess, Ilu).

Supplementary data 7. Enrichment of positive selected genes (PSGs) in each common ancestral branch of luminous family (AB-LRSE, AB-LRS, AB-LR, AB-Lam, AB-Ela) and luminous species (Sta, Ess, Ilu).

Supplementary data 8. Gene number of acyl-CoA synthetase (ACS) superfamily identified in beetles.

Supplementary data 9. Information of acyl-CoA synthetase (ACS) superfamily in beetles.

Supplementary data 10. Information of luciferase (Luc) and luciferase-like (LL) genes in Fig. 3A.

Supplementary data 11. Distribution of luciferase (Luc) and surrounding genes at

contigs or scaffolds or chromosome in seven luminous beetles.

Supplementary data 12. Distribution of luciferase (Luc) and luciferase-like (LL) gene family (LLL) at contigs or scaffolds or chromosome in beetles.

Supplementary data 13. Gene number in the gene family including candidate enzymes involved in the luciferin metabolic pathway.

Supplementary data 14. Information of enzyme-encoding genes involved in the luciferin metabolic pathway.

Supplementary data 15. Amino acids and posterior probabilities of ancestral luciferases.
